# Molecular portraits of cell cycle checkpoint kinases in cancer evolution, progression, and treatment responsiveness

**DOI:** 10.1126/sciadv.adf2860

**Published:** 2023-06-30

**Authors:** Elena Oropeza, Sinem Seker, Sabrina Carrel, Aloran Mazumder, Daniel Lozano, Athena Jimenez, Sabrina N. VandenHeuvel, Dillon A. Noltensmeyer, Nindo B. Punturi, Jonathan T. Lei, Bora Lim, Susan E. Waltz, Shreya A. Raghavan, Matthew N. Bainbridge, Svasti Haricharan

**Affiliations:** ^1^ Aging and Cancer Immunology, Sanford Burnham Prebys Medical Discovery Institute, La Jolla, CA, USA.; ^2^ NCI-designated Cancer Center, Sanford Burnham Prebys Medical Discovery Institute, La Jolla, CA, USA.; ^3^ Lester and Sue Smith Breast Cancer Center, Baylor College of Medicine, Houston, TX, USA.; ^4^ Department of Oncology/Medicine, Baylor College of Medicine, Houston, TX, USA.; ^5^ Department of Cancer Biology, University of Cincinnati, Cincinnati, OH, USA.; ^6^ Research Service, Cincinnati Veteran's Affairs Medical Center, 3200 Vine St., Cincinnati, OH, USA.; ^7^ Texas A&M University, College Station, TX, USA.; ^8^ Rady Children’s Institute for Genomic Medicine, San Diego, CA, USA.

## Abstract

Cell cycle dysregulation is prerequisite for cancer formation. However, it is unknown whether the mode of dysregulation affects disease characteristics. Here, we conduct comprehensive analyses of cell cycle checkpoint dysregulation using patient data and experimental investigations. We find that *ATM *mutation predisposes the diagnosis of primary estrogen receptor (ER)^+^/human epidermal growth factor (HER)2^−^ cancer in older women. Conversely, CHK2 dysregulation induces formation of metastatic, premenopausal ER^+^/HER2^−^ breast cancer (*P* = 0.001) that is treatment-resistant (HR = 6.15, *P* = 0.01). Lastly, while mutations in *ATR *alone are rare,* ATR*/*TP53* co-mutation is 12-fold enriched over expected in ER^+^/HER2^−^ disease (*P* = 0.002) and associates with metastatic progression (HR = 2.01, *P* = 0.006). Concordantly, ATR dysregulation induces metastatic phenotypes in *TP53 *mutant, not wild-type, cells. Overall, we identify mode of cell cycle dysregulation as a distinct event that determines subtype, metastatic potential, and treatment responsiveness, providing rationale for reconsidering diagnostic classification through the lens of the mode of cell cycle dysregulation..

## Teaser

While all cells must dysregulate the cell cycle to transform into cancer, it turns out that it really matters how they do it..

## INTRODUCTION

Ataxia-telangiectasia mutated (ATM)/Checkpoint kinase (CHK)2 and Ataxia telangiectasia and Rad3-related protein (ATR)/CHK1 are cell cycle checkpoint complexes activated by DNA damage (*1*, *2*). Although roles for these proteins in regulating cell cycle progression are complex and often redundant, they can be generalized as ATM/CHK2 and ATR/CHK1 inhibit the cell cycle at G_1_-S and G_2_-M phases, respectively (*1*, *3*, *4*). In cases where prolonged cell cycle arrest is insufficient for DNA repair, these kinases trigger cell death through p53-dependent and -independent mechanisms (*5*–*8*). The ATR checkpoint is also responsive to replication stress, one of the hallmarks of cancer (*9*). Overall, loss of these checkpoint kinases contribute to genomic instability (*10*). Consequently, these cell cycle checkpoint kinases are important tumor suppressors across cancer types.

Breast cancer is one of the most frequently diagnosed cancers globally, and therefore, one of the most common causes of cancer-related death (*11*). Estrogen receptor (ER) status of breast cancer stratifies diagnoses as ER positive (ER^+^) and negative (ER^−^) (*12*). Amplification and/or mutation of the tyrosine kinase receptor human epidermal growth factor (*HER2*) further categorizes breast cancer subtypes as HER2^+^ or HER2^−^ (*13*). ER^+^/HER2^−^ breast cancer is far more common than any other breast cancer subtype and predicts response to endocrine therapies that inhibit ER signaling (*14*, *15*). However, ~20% of patients with ER^+^/HER2^−^ breast cancer are intrinsically resistant to endocrine therapy and ~40% of patients with ER^+^/HER2^−^ breast cancer acquire resistance over time (*14*, *16*, *17*). Breast cancers characterized by amplification of HER2 are aggressive but treatable with therapies targeting HER2 activity (*18*). Triple-negative breast cancer (TNBC)—which is characterized by lack of substantial expression of ER, its downstream effector progesterone receptor (PR), and HER2—has few available targeted therapeutics, is highly metastatic, and associates with poor patient outcome (*12*). Understanding molecular contributors to evolution of treatment-resistant ER^+^/HER2^−^ and to TNBC is critical for improving clinical diagnostics and therapeutics.

The importance of cell cycle dysregulation for breast cancer evolution is well established (*19*, *20*). Large and multiple independent epidemiological studies demonstrate association of germline variants in *ATM* and *CHEK2* with incidence of ER^+^/HER2^−^ breast cancer (Table 1) (*21*–*25*). Somatic failure to activate ATM/CHK2, through loss of upstream DNA repair signaling, such as the MutL complex of the mismatch repair pathway, in ER^+^/HER2^−^ breast cancer also induces resistance to standard endocrine therapies (*26*–*28*). Other studies demonstrate significant associations between high levels of phospho-ATM and heightened responsiveness to endocrine therapy (*29*–*31*). To date, however, there is a lack of understanding of whether early dysregulation of ATM/CHK2 predisposes formation of breast cancer that is treatment resistant or aggressively metastatic. Even less is known about the association of *ATR/CHEK1* mutations, either germ line or somatic, with breast cancer incidence or outcome. There is evidence that germline mutations in *ATR* are enriched in patients with familial breast cancer and that ATR/CHK1 may serve as therapeutic targets in TNBCs (Table 1) (*32*–*34*). However, whether mutations in *ATR*/*CHEK1* contribute to evolution of specific subtypes of breast cancer or to disease progression remains uncertain (*35*, *36*).

**Table 1.** Breast cancer subtypes and known associations with cell cycle checkpoint kinase dysregulation based on epidemiological studies in the literature.

Breast cancer subtype	Receptor status	Cell cycle checkpoint kinase association	Reference citation
Luminal A	ER^+^ PR^+^ HER^−^	*ATM* germline variant	(*78*, *79*)
		*CHEK2* germline variant	(*78*–*82*)
		CHK2 somatic dysregulation	(*26*)
Luminal B	ER^+^ PR^+^ HER2^+^	*ATM* germline variant	(*83*, *84*)
		*CHEK2* germline variant (truncating)	(*81*)
		*TP53* mutation	(*78*, *79*)
HER2-enriched	ER^−^ PR^−^ HER2^+^	*CHEK2* germline variant	(*79*)
		*TP53* mutation	(*78*)
Basal-like	ER^−^ PR^−^ HER2^−^	*TP53* mutation	(*78*, *80*)
		*ATR* germline variant	(*85*)
		ATR somatic dysregulation	(*85*)
Normal-like	ER^−^ PR^−^ HER2^−^ CK5^−^ EGFR^−^	Unknown	

Overall, the utility of cell cycle checkpoint kinase dysregulation as prognostic markers of disease severity or as predictors of treatment response remains undefined. Previous studies in these areas have suffered from conflicting or inconclusive results partly due to a lack of adequate sample size in patient datasets and an absence of experimental validation (*25*, *37*, *38*). Cell cycle checkpoint dysregulation occurs early in tumor evolution, and there are several ways of achieving this end. Understanding whether specific cell cycle dysregulation events determine evolution and clinical outcome of different cancer subtypes is critical for identifying the potential of each cell cycle protein as a prognostic/predictive biomarker and even as a therapeutic target (*25*, *39*). Here, using breast cancer as a model, we undertake a systematic evaluation of the relative contribution of dysregulation of each cell cycle checkpoint kinase to the formation of tumors of distinct subtypes, metastatic progression, and treatment responsiveness with a range of informatic and experimental approaches as described below.

## RESULTS

### Mutation of specific cell cycle checkpoint kinase genes promotes the evolution of distinct breast cancer subtypes.

**Fig. 1.**
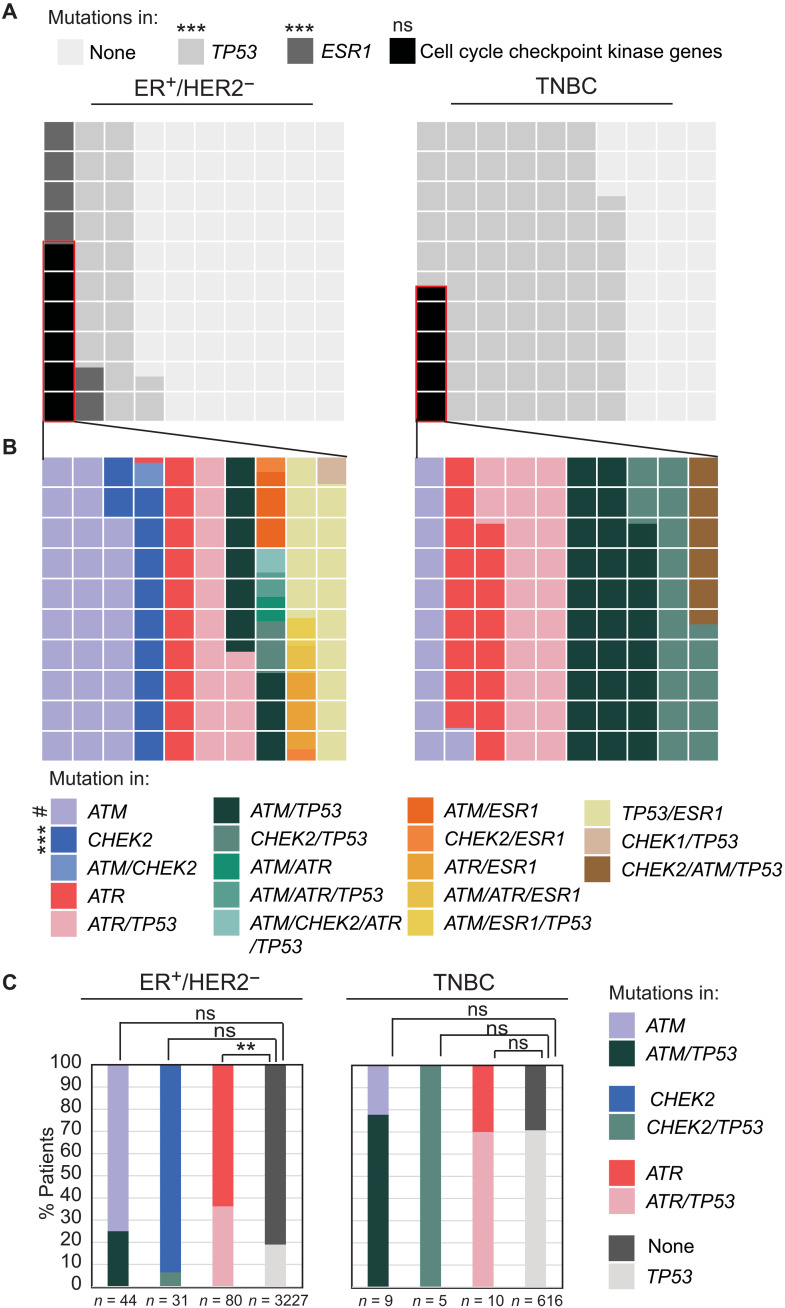
Mutational frequency of cell cycle checkpoint kinase genes differs across breast cancer subtype. (**A** and **B**) Waffle charts showing control (*ESR1* and *TP53*) and cell cycle checkpoint kinase (*ATM*, *ATR*, *CHEK1*, and *CHEK2*) gene mutational frequencies in ER^+^/HER2^−^ versus TNBC. Each square represents 1% mutational frequency. (**C**) Stacked column graphs quantifying incidence of mutations in *ATM*/*ATR*/*CHEK2* with and without mutations in *TP53* in indicated breast cancer subtypes. Fisher’s exact test determined the *P* values that were adjusted for multiple comparison using the Holm’s method. Dataset composition is presented in fig. S1A. #*P* ≤ 0.1, **P ≤ 0.01, and ***P ≤ 0.001. ns, not significant.

Using a meta-dataset composed of six independent studies (Fig. 1 and fig. S1A), we compared frequency of mutations, both germ line and somatic, in each of the four cell cycle checkpoint kinase genes *ATM*, *CHEK2*, *ATR*, and *CHEK1* in ER^+^/HER2^−^ and TNBC samples. ER^+^/HER2^−^ samples were excluded from analyses because of insufficient sample size. We included known cancer drivers *ESR1* and *TP53* as positive controls for mutational frequency in ER^+^/HER2^−^ and TNBC, respectively. As expected, *ESR1* mutations are more common, and *TP53* mutations are less common in ER^+^/HER2^−^ than in TNBC tumors (*P* < 1.3 × 10^−15^ each) (Fig. 1A). The cumulative frequency of mutation incidence in all four cell cycle kinase genes is comparable between ER^+^/HER2^−^ and TNBC samples (Fig. 1A). On an individual gene level, we observed >5-fold enrichment for mutations in *CHEK2* (29 of 3382 versus 0 of 640, *P* = 0.001 after adjustment for multiple comparisons) in ER^+^/HER2^−^ breast cancer samples relative to TNBCs, but no statistically significant enrichment for *ATM*, *ATR*, or *CHEK1* mutations in either subtype (Fig. 1B). *CHEK1* mutation is extremely rare in both ER^+^/HER2^−^ and TNBC and was, therefore, excluded from further analyses. Although mutation of *ATR* alone is not enriched in either ER^+^/HER2^−^ or TNBC, we found twofold enrichment for comutation of *ATR* and *TP53* in ER^+^/HER2^−^ breast cancer (36% *ATR*/*TP53* comutated versus 18% *TP53* mutation alone, *P* = 0.002) (Fig. 1C). Results from this overview analysis indicate that *CHEK2* is the only cell cycle checkpoint kinase that robustly correlates with the evolution of a specific breast cancer subtype, i.e., ER^+^/HER2^−^, when mutated.

#### CHEK2 mutation enriches for diagnosis of premenopausal ER^+^/HER2^−^ breast cancer

**Fig. 2.**
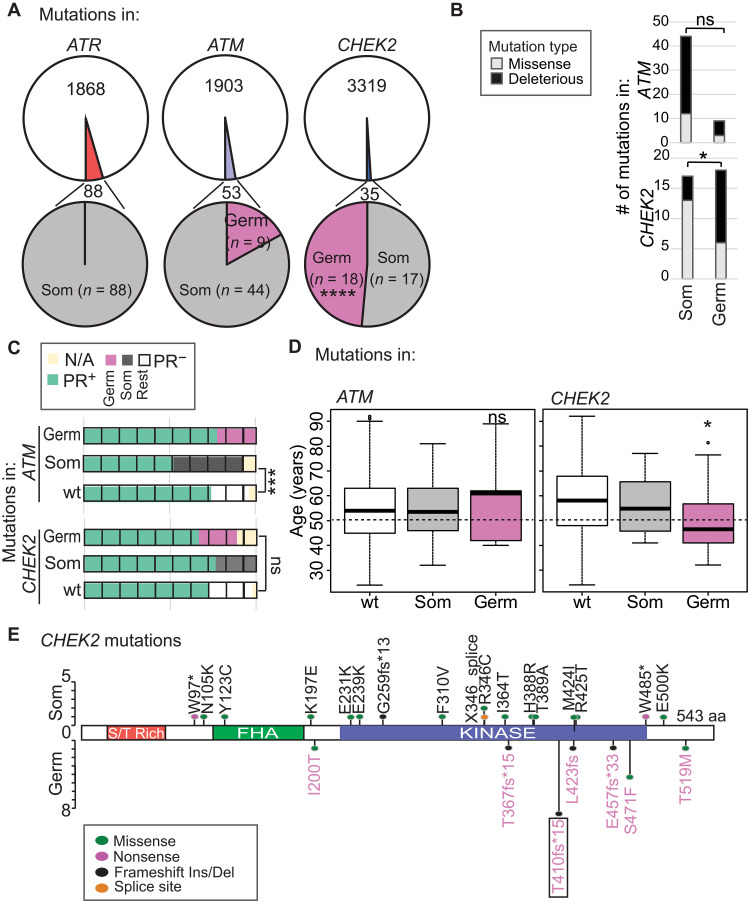
Germline mutations in *CHEK2* drive associations with younger age at diagnosis of ER^+^/PR^+^/HER2^−^ breast cancer. (**A**) Pie charts showing proportion of *ATR*, *ATM*, and *CHEK2* mutations that are somatic and germline in patients with ER^+^/HER2^−^ breast cancer. (**B**) Stacked columns demonstrating number of *CHEK2* and *ATM* mutations that are missense and deleterious (frameshift, nonsense, or splice site) based on their somatic (Som) or germline (Germ) status in ER^+^/HER2^−^ breast cancer samples. (**C**) Waffle chart depicting percent ER^+^/HER2^−^ breast cancer samples with indicated mutations categorized by PR status (n/a; PR status not available). (**D**) Box plot indicating median age at diagnosis for indicated groups of patients with breast cancer. Error bars describe SD. Dotted line indicates average age at menopause for women in the United States. (**E**) Lolliplot of all observed mutations in *CHEK2*. Box indicates the mutation studied experimentally in subsequent analyses. aa, amino acid. Fisher’s exact test (A) to (C) and two-tailed independent sample Student’s *t* test (D) determined *P* values. *P ≤ 0.05, ***P ≤ 0.01, and ****P ≤ 0.001. wt delineates patients with no mutations in genes of interest.

The predisposition toward developing ER^+^/HER2^−^ breast cancer from cells with *CHEK2* mutations indicated the potential involvement of germline variants. As previously established (*21*), *ATM* and *CHEK2* are mutated in the germ line, while *ATR* is not (Fig. 2A). We found that *CHEK2* is the only cell cycle checkpoint kinase gene that is more likely to be mutated in the germ line than somatically (threefold enrichment in ER^+^/HER2^−^ breast cancer relative to *ATM* and >50-fold enrichment relative to *ATR*, *P* = 3.7 × 10^−12^) (Fig. 2A). We compared the landscape of germline and somatic *CHEK2* mutations and found threefold enrichment for deleterious (nonsense, frameshift, or splice site) mutations in the germline group (12 of 18 versus 4 of 18 somatic, *P* = 0.02), but no such enrichment among *ATM* mutations (*P* = 0.13) (Fig. 2B). We next tested whether germline mutations in *CHEK2* alter PR positivity, because ER^+^/HER2^−^ tumors can be either PR^+^ (strongly driven by ER signaling) or PR^−^ constituting distinct breast cancer subtypes that coincide with Luminal A and B subtypes, respectively (see Table 1) (*12*). We found that neither germline nor somatic mutations in *CHEK2* affect PR positivity, with tumors remaining predominantly PR^+^ as is the case with wild-type *CHEK2* tumors (Fig. 2C). Conversely, we observed that somatic mutations in *ATM* associate with twofold enrichment for PR negativity relative to either *ATM* wild-type or germline–mutated tumors (*P* = 0.002) (Fig. 2C).

Last, we assessed association of somatic and germline mutations in *CHEK2* and *ATM* with age at diagnosis. We found that germline, but not somatic, mutations in *CHEK2* associate strongly with diagnosis of premenopausal ER^+^/HER2^−^ disease: Median age for *CHEK2* germline carriers is 46 years, while that of *CHEK2* somatic or wild-type patients is 55 and 58 years, respectively (*P* = 0.02) (Fig. 2D). Conversely, mutations in *ATM* do not affect age at diagnosis: Patients with germline or somatic *ATM* mutations remain postmenopausal with median ages of 61 and 54 years, respectively, as expected for patients with ER^+^/HER2^−^ breast cancer (Fig. 2D). Overall, these data suggest that germline mutations in *CHEK2* contribute to the evolution of ER-driven, premenopausal ER^+^/HER2^−^ breast cancer.

#### CHEK2 mutation induces the evolution of ER^+^ premalignant growth in a genetically engineered mouse model

**Fig. 3.**
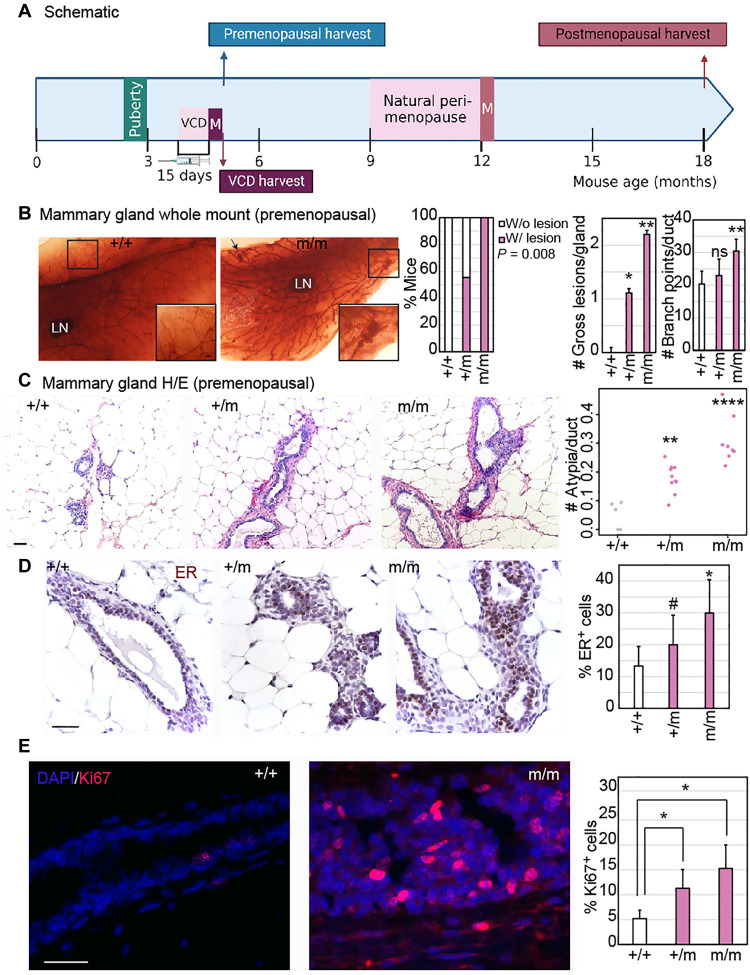
*CHEK2* mutation induces formation of ER^+^ preneoplastic lesions in the mouse mammary gland. (**A**) Schematic of experimental design. VCD, 4-vinylcyclohexene diepoxide; M, menopause. (**B**) Representative images of whole-mounted mammary glands (1.5×) with cleared fat pads showing mammary ductal structure, which was used to quantify the incidence (w/, with; w/o, without) and the number of gross (macroscopic) mammary lesions (representative ×6 magnification shown in inset) and the number of branch points in mammary ducts, represented in bar graphs. Statistical differences in incidence of lesions were tested using Fisher’s exact test and in number of lesions and branch points using a two-tailed Student’s *t* test. (**C** to **E**) Representative images and accompanying bar graph quantification of the number of microscopic atypia using hematoxylin and eosin (H&E) staining (C), percent atypical cells that are ER^+^ by immunohistochemistry (D), and percent proliferating cells using immunofluorescence for Ki67 (E). Scale bars, 20 μm. Two-tailed Student’s *t* test derived all *P* values. For all panels, wild-type (+/+), heterozygous (+/m), and homozygous (m/m) CHEK2*1100delC mice were harvested at 20 weeks (5 months) of age. Error bars in all bar graphs represent SD. #*P* ≤ 0.1, *P ≤ 0.05, **P ≤ 0.01, and ***P ≤ 0.001. Associated data validating results in the FVB background are presented in fig. S2. DAPI, 4′,6-diamidino-2-phenylindole.

To experimentally test whether germline *CHEK2* variants promote evolution of ER^+^ cancer in the premenopausal breast, we used a genetically engineered mouse model expressing the CHEK2*1100delC variant (p. Thr410fs*15) (*40*), the most common variant in our study (Fig. 2E). Using this mouse model, we investigated the impact of CHK2 loss on tumor evolution in young and old mice and after experimental induction of menopause (Fig. 3A). In the mammary gland, whole mounts from premenopausal (5-month-old) C57/B6 female mice, we identified formation of macroscopic, preneoplastic mammary lesions in 100% of mice homozygous for the mutant allele (*n* = 5 of 5), 56% of mice heterozygous for the mutation (*n* = 5 of 9), and 0% of mice who were wild-type (*n* = 0 of 5) (*P* = 0.008) (Fig. 3B).

Homozygous mutant mice also have multiple macroscopic lesions per mammary gland (*P* = 0.0004) and heavy side branching (Fig. 3B), indicators of unchecked proliferation, although ductal length is unperturbed compared to wild-type mice (fig. S2A). These findings are supported by an observed increase in microscopic, atypical lesions in the mammary ducts of homozygous (sixfold increase over wild-type, *P* = 1.7 × 10^−5^) and heterozygous (fourfold increased over wild-type, *P* = 0.001) mutant mice (Fig. 3C). Because mouse strain backgrounds can influence mammary phenotypes, we confirmed these phenotypes in an FVB background, reproducing the increase in macroscopic preneoplastic lesions (*P* = 0.04) (fig. S2B) and microscopic atypia (*P* = 0.002) (fig. S2C) in homozygous mutants relative to wild-type controls. Preneoplastic lesions in homozygous mutant mice in both backgrounds are also ER^+^ (Fig. 3D and fig. S2D) and PR^+^ (fig. S2, E and F) with a twofold higher rate of median positivity than wild-type ducts (*P* = 0.02 for ER and *P* = 0.007 for PR) and highly proliferative (Fig. 3E and fig. S2G). Preneoplastic lesions in homozygous mutant mice also demonstrate the expected increase in levels of DNA damage (fig. S2, H and I) and associated apoptosis (fig. S2, J and K) relative to normal ducts in wild-type controls.

#### Mutant CHEK2-induced premalignant cancer evolution is suppressed in the postmenopausal mammary epithelium

**Fig. 4.**
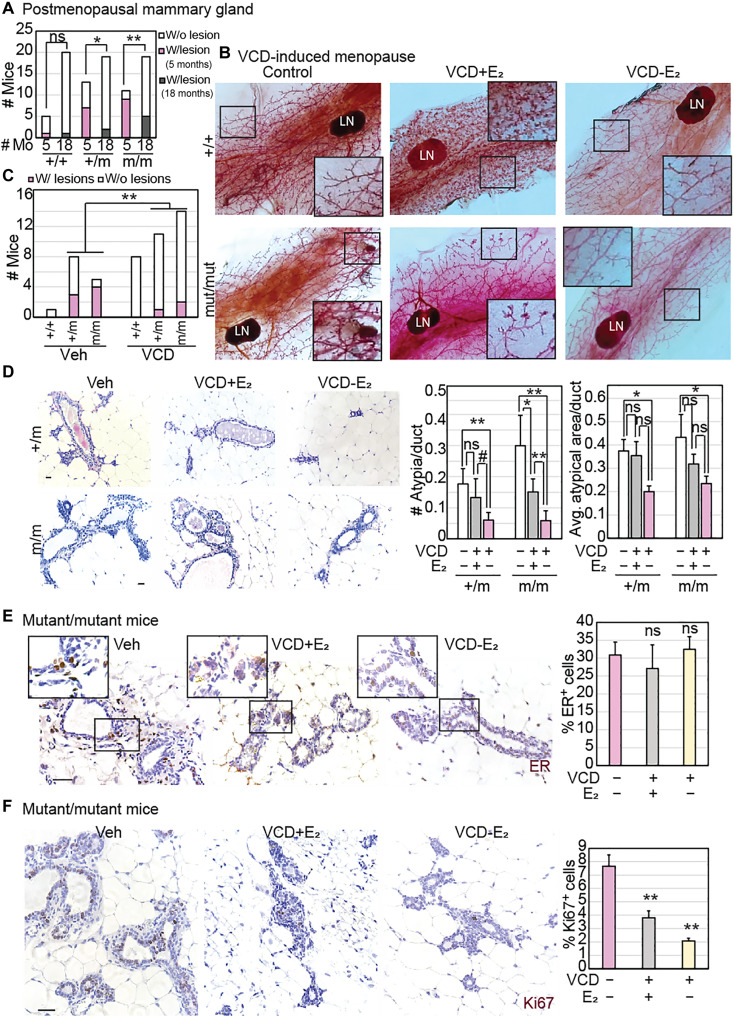
*CHEK2* mutation induces formation of highly proliferative mammary lesions preferentially in premenopausal mice. (**A**) Stacked column graph representing the proportion of wild-type (+/+), heterozygous (+/m), and homozygous (m/m) CHEK2*1100delC mutant female mice at 5 (premenopausal) and 18 (postmenopausal) months of age. (**B** and **C**) Representative images (B) of whole-mounted mammary glands (1.5×; inset magnification, ×6) from mice from each specified genotype, treated with or without VCD, with cleared fat pads showing mammary ductal structure that was used to quantify the incidence of gross (macroscopic) mammary lesions, represented as a stacked column graph (C). Statistical differences in incidence of lesions tested using Fisher’s exact test. LN, lymph node. (**D** to **F**) Representative images and accompanying bar graph quantification of the number of microscopic atypia using H&E staining (D), percent atypical cells that are ER^+^ by immunohistochemistry [(E); inset magnified, ×2], and percent proliferating cells using immunofluorescence for Ki67 (F) under the specified treatment conditions (Vehicle, Veh; VCD; E_2_, β-estradiol). Scale bars, 20 μm. Two-tailed Student’s *t* test determined *P* values. Mice were harvested at 20 weeks (5 months) of age for (B) to (F). Whiskers in all bar graphs represent SD. #*P* ≤ 0.1, *P ≤ 0.05, and **P ≤ 0.01. Associated data are presented in fig. S3.

To investigate whether mutant *CHEK2*-induced premalignant growth is altered by either age or menopausal status, we first compared premenopausal (5-month-old) and postmenopausal (18-month-old) mammary glands from heterozygous and homozygous CHEK2*1100delC mutant female mice. We found that the mammary epithelia of 18-month-old mutant mice harbor significantly fewer macroscopic lesions (Fig. 4A and fig. S3A) than their 5-month-old counterparts, suggesting that the postmenopausal mammary environment suppresses premalignant evolution. As a more direct test of the impact of the postmenopausal mammary environment on *CHEK2* mutation–induced breast cancer evolution, we used the 4-vinylcyclohexene diepoxide (VCD) model (*41*) to induce menopause in heterozygous and homozygous mutant, female mice. Mice were either administered VCD or placebo injections for 15 days and euthanized 6 weeks after administration at 5 months of age (schematic in Fig. 3A).

As expected, we observed almost complete suppression of serum estradiol levels within 4 weeks of administration of VCD (*P* = 0.002) in a manner comparable to that seen in postmenopausal mice (fig. S3B). We observed macroscopic preneoplastic mammary lesions in 80% of the vehicle-treated homozygous mutant mice (4 of 5) and 40% of heterozygous mice (3 of 8) compared to only 14% (2 of 14) and 9% (1 of 11) of VCD-treated controls (cumulative *P* = 0.005) (Fig. 4, B and C). The decrease in preneoplastic incidence is echoed at the microscopic level with 75 and 85% decrease in lesion incidence in heterozygous and homozygous mutants, respectively (*P* = 0.001) (Fig. 4D). Existing atypia in VCD-treated mutant mice also demonstrated significant shrinkage in the area (Fig. 4D). The number of ER^+^ cells in atypia in VCD-treated mice remains comparable to that in vehicle-treated controls, although levels of ER appeared lower (Fig. 4E). Irrespective of ER positivity, proliferation in preneoplastic cells is significantly suppressed in VCD-treated homozygous mutants compared to vehicle-treated controls (Fig. 4F).

To test whether suppression of preneoplastic evolution in postmenopausal *CHEK2* mutant mammary glands is due to loss of mitogenic estrogen stimuli caused by suppression of ovarian function after menopause, we included a control group of mice, whose drinking water was supplemented with estradiol during and after administration of VCD. We found that estrogen supplementation robustly induced side branching in the wild-type mice but was only partially effective in the homozygous mutant mammary glands (Fig. 4B). Similarly, estrogen supplementation only partially rescues the preneoplastic phenotype in VCD-treated mice and lesions in estrogen-supplemented VCD-treated mice have a distinct morphology from that seen in vehicle-treated premenopausal mice (Fig. 4D). Together, these data suggest that the perceived rescue of macroscopic lesions might be the effect of estrogen stimulation independent of CHK2 activity. In support of this hypothesis, the proliferative inhibition caused by VCD-induced menopause is not rescued by estrogen supplementation (*P* = 0.0005) (Fig. 4F).

Because CHEK2*1100delC mice have indolent tumorigenesis and only a small proportion present with mammary tumors, we next examined evolution of preneoplastic lesions in a mammary tumor susceptible transgenic mouse model (MMTV-Ron kinase) crossed into the CHEK2*1100delC line (*42*). One hundred percent of the double transgenic mice acquire tumors by 1 year of age (i.e., premenopausally) (*42*). Analysis of preneoplastic growth in mammary glands of double transgenic relative to single transgenic (MMTV-Ron alone) mice, demonstrated that presence of the CHEK2*1100delC mutation drives aggressive preneoplastic growth in young mice (9 to 10 months old) but not in old mice (14 to 15 months olds) (fig. S3C). This is in sharp contrast to the intuitively expected trajectory observable in MMTV-Ron mice without the *CHEK2* mutation, where preneoplastic growth increases in number and size with age (fig. S3C). Together, these data suggest a causal and age-specific role for CHK2 loss in premenopausal ER^+^ breast cancer evolution that is only partially affected by estrogen-mediated mitogenesis.

### Mutation of individual cell cycle checkpoint kinase genes distinctly impacts disease progression

**Fig. 5.**
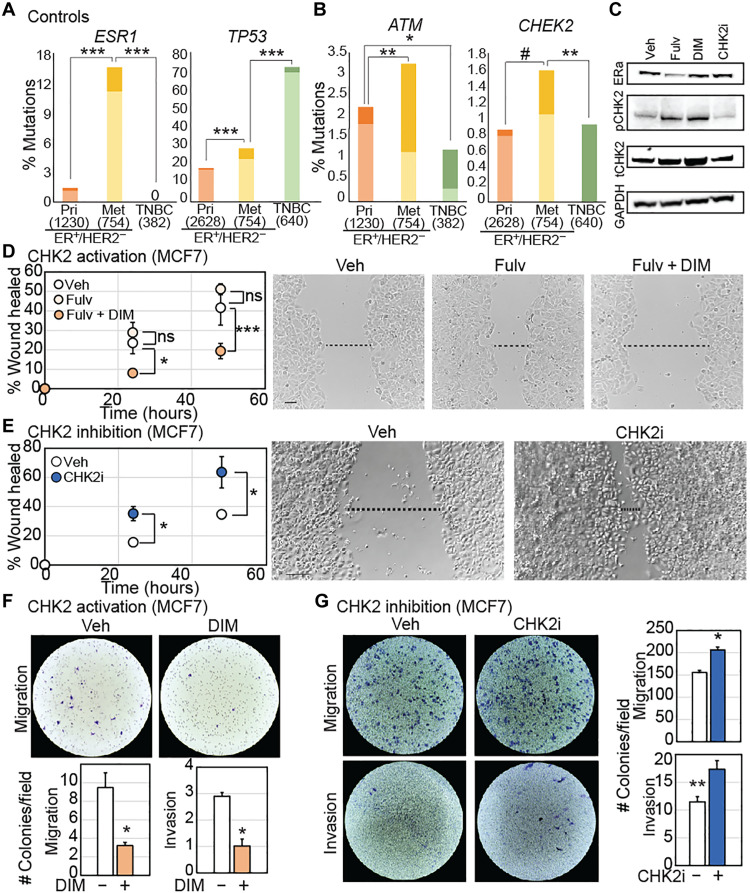
CHK2 loss promotes metastatic phenotypes in ER^+^/HER2^−^ breast cancer cells. (**A** and **B**) Stacked columns representing frequency of mutations in *ESR1* and *TP53* (A, control genes) and *ATM* and *CHEK2* (B) in ER^+^/HER2^−^ primary (Pri) versus metastatic (Met) breast cancer and TNBC. Darker shading indicates incidence of mutations in multiple genes of interest, while lighter shading indicates mutation of only the specified gene of interest. *P* values were derived by comparing light shaded columns between the three breast cancer subtypes in a Fisher’s exact test. Holm’s adjustment for multiple comparisons was conducted. Sample sizes in parentheses. (**C**) Western blots demonstrating impact of indicated inhibitors and activators on phosphorylation of target proteins in MCF7 cells. (**D** and **E**) Representative images of wound healing assay of ER^+^/HER2^−^ breast cancer cell line, MCF7, treated with vehicle (Veh), 100 nM Fulvestrant (Fulv), or the combination of 10 μM DIM, a CHK2 activator and fulvestrant (D), or 100 nM CHK2 inhibitor dihydrate (CHK2i) (E) at 48 hours. Dot plots representing quantification of area of scratch at 0, 24, and 48 hours with error bars depicting SD. (**F** and **G**) Representative images of transwell migration (top) and invasion (G, bottom) assays at 48 hours after treatment with vehicle, DIM, or CHK2i, along with bar graphs depicting quantification. DIM treatments were done in media with charcoal-stripped serum + 10 pM β-estradiol, while CHK2i assays were done in media with full serum. Error bars represent SD. Two-tailed Student’s *t* test determined *P* values for (D) to (G). #*P* ≤ 0.1, *P ≤ 0.05, **P ≤ 0.01, and ***P ≤ 0.001. Supporting data validating activity of compounds used and independent confirmation in a second cell line are presented in fig. S4.

We next tested whether mutations in cell cycle checkpoint kinase genes modulate metastatic progression in breast cancer patients. As expected, in our breast cancer meta-dataset, incidence of *ESR1* mutations is highly enriched, and of *TP53* is moderately enriched, in metastatic ER^+^/HER2^−^ breast cancer compared to primary (Fig. 5A) (*43*, *44*). Among cell cycle checkpoint kinase genes, we observed 1.5-fold enrichment for *ATM* mutation (alone, not in combination with any other gene of interest) in primary ER^+^/HER2^−^ breast cancer relative to metastatic disease (*P* = 0.003) (Fig. 5B). Strikingly, *ATM* is the only cell cycle checkpoint kinase gene enriched for mutation in the primary setting, as both *CHEK2* (*P* = 0.001; Fig. 5B) and *ATR* mutations (*P* = 0.0002) are enriched in metastatic disease.

#### CHK2 dysregulation causally affects metastasis in breast cancer cells

To understand the functional relevance of CHK2 dysregulation in promoting the metastasis of ER^+^/HER2^−^ cancer, we experimentally dysregulated CHK2 in two independent cell lines [MCF7 (Fig. 5) and T47D (fig. S4)]. First, we activated CHK2 exogenously using fulvestrant, a standard endocrine therapy that induces modest CHK2 activation (Fig. 5C and fig. S4A), and di-indolyl methane (DIM), a robust, small-molecule CHK2 activator (*45*, *46*) (Fig. 5C and fig. S4, A and B). In wound healing assays, treatment with endocrine therapy alone is not sufficient to impact cell migration but administration of DIM in addition to endocrine therapy significantly inhibits motility [MCF7 (19% versus 42% wound healed, *P* = 0.008; Fig. 5D) and T47D (6% versus 32%, *P* = 0.01; fig. S4C)]. Conversely, inhibition of CHK2 using a small-molecule inhibitor, CHK2 inhibitor dihydrate (*47*) significantly promotes motility in wound healing assays in both cell lines [MCF7 (63% versus 45% wound healed, *P* = 0.01; Fig. 5E) and T47D (50% versus 33%, *P* = 0.02; fig. S4D)]. These observations were orthogonally replicated through assessment of migration and invasion in transwell assays in both MCF7 and T47D cells (fig. S4, E and F).

To rule out confounding effects of endocrine treatment on motility and invasiveness, wound healing, transwell migration, and invasion assays were also conducted with either DIM or CHK2 inhibitor treatment alone, without addition of endocrine therapies. Results from these experiments confirmed the role of CHK2 activation in inhibiting migration and invasion (Fig. 5F and fig. S4G) and of CHK2 inhibition in promoting these phenotypes (Fig. 5G and fig. S4H), respectively, in each of these assays.

**Fig. 6.**
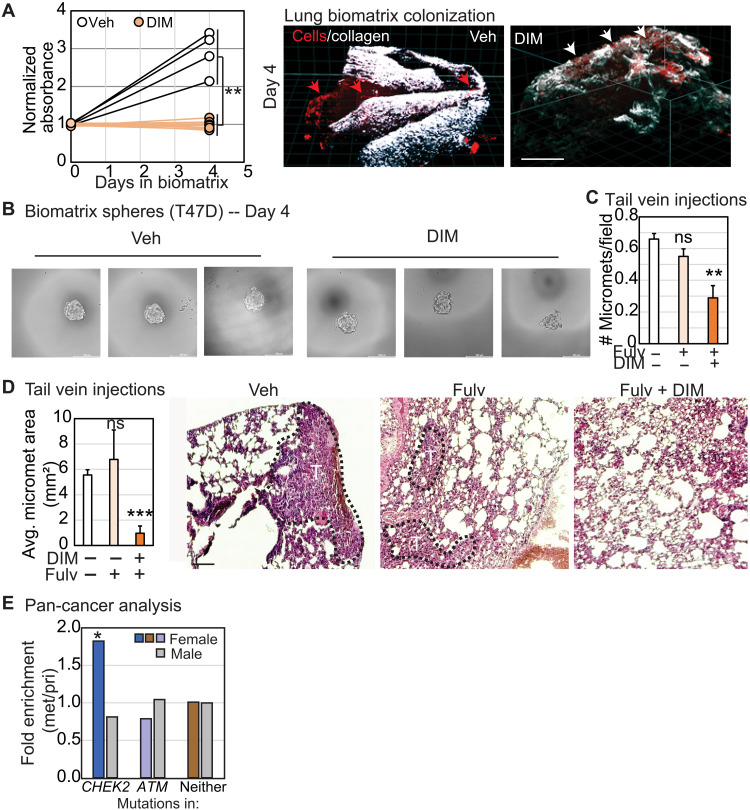
CHK2 loss promotes metastatic cancer. (**A**) Dot plots quantifying cell clusters that invade into the bioengineered lung matrix (collagen) and establish micrometastatic colonies along with representative images showing RFP-tagged T47D cell clusters and the collagen matrix (white). Micrometastatic colonies established after invasion into the collagen matrix are indicated by red arrows, and cell clusters that remain above the collagen unable to invade into the matrix are indicated by white arrows. Scale bars, 200 μm. (**B**) Representative images demonstrating that T47D hanging drop array spheroids maintain viability after DIM administration. (**C** and **D**) Bar graphs describing quantification of average number (C) and area (D) of micrometastases (tumors, T) in the lungs of mice administered T47D cells through tail vein injections under the indicated treatment conditions, identifiable as in representative images 6 weeks after tail vein injections of T47D cells into nude mice. Scale bars, 50 μm. (**E**) Column graph representing fold enrichment for *CHEK2* and *ATM* mutations in metastatic samples relative to primary samples in a pan-cancer analysis of MSKCC data, contextualized by gender of the patient. Two-tailed Student’s *t* test determined *P* values for (A) to (D) and Pearson Chi-square test for (E). *P ≤ 0.05, **P ≤ 0.01, and ***P ≤ 0.001.

Next, we seeded red fluorescent protein (RFP)–tagged T47D spheroids into bioengineered porcine lung biomatrix scaffolds. We visualized and quantified the ability of these cells to invade and colonize the lung matrix and establish cell clusters representing micrometastatic colonies. We observed establishment of micrometastatic colonies within 4 days in vehicle-treated T47D cells, while this ability is completely abrogated by treatment with DIM (Fig. 6A; 2.8-fold increase in absorbance relative to day 0 in control versus no increase in DIM-treated cells, *P* = 0.006). DIM treatment did not significantly affect spheroid growth outside the biomatrix (Fig. 6B), suggesting a selective impact of CHK2 activation on the ability of cells to invade and establish themselves at distant organs. Last, we confirmed these observations in vivo by conducting tail vein injections with T47D cells in nude mice randomized to three different groups, vehicle, fulvestrant, and combinatorial DIM and fulvestrant treatment. We examined lungs for micrometastases 6 weeks after treatment and found significant inhibition in both number and size of micrometastases in the combination-treated group relative to vehicle and fulvestrant treated control animals (Fig. 6, C and D). As with CHEK2*1100delC mutation bearing mice (fig. S2, H and I), inhibiting CHK2 in these cells also induces significant levels of DNA damage as evidenced by 53BP1 and γH2AX nuclear foci (fig. S4I), although the causality of this DNA damage to the metastatic phenotype is uncertain. The association of *CHEK2* mutation with metastasis appears paradigmatic but gender dependent: A pan-cancer analysis across >10 different cancer types demonstrated 1.8-fold enrichment for *CHEK2* (*P* = 0.045), but not *ATM*, mutations in metastatic relative to primary tumors only in women, not in men (Fig. 6E).

#### ATR dysregulation causally impacts metastasis in TP53 mutant breast cancer cells

**Fig. 7.**
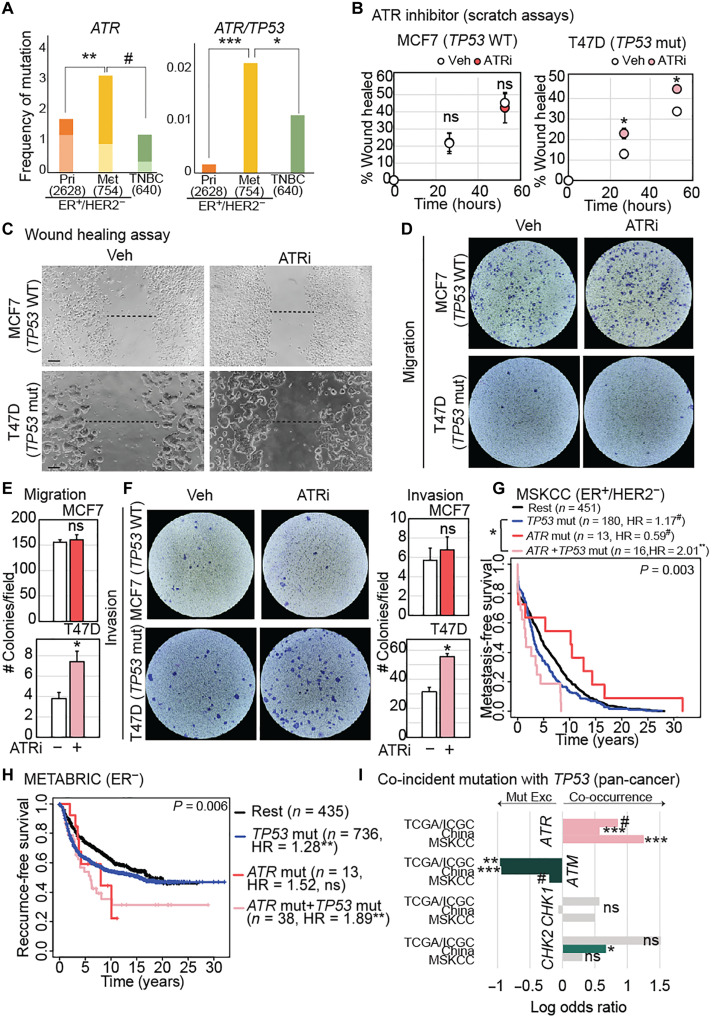
ATR dysregulation promotes metastatic breast cancer dependent on *TP53* status. (**A**) Bar graphs representing frequency of *ATR* and *TP53* mutations in ER^+^/HER2^−^ primary versus metastatic breast cancer and in TNBC. The darker shading indicates that there were mutations in multiple genes of interest, while the lighter shading indicates that only the specified gene of interest was mutated in those samples. *P* values were derived by comparing the light shaded columns between the three breast cancer subtypes. Sample sizes in parentheses. (**B** and **C**) Representative images (C) of wound healing assay of ER^+^/HER2^−^ breast cancer cell lines, MCF7 (*TP53* wild-type; wt), and T47D (*TP53* mutant; mut), treated with vehicle (Veh) or ATR inhibitor (ATRi) at 48 hours. Dot plots representing quantification of area of scratch at 0, 24, and 48 hours with error bars depicting SD (B). (**D** to **F**) Representative images of transwell migration (D) and invasion (F) assays at 48 hours after treatment with vehicle or ATRi, along with bar graphs depicting quantification (E) and (F). Error bars represent SD. (**G** and **H**) Kaplan-Meier survival curves representing metastasis-free (G) and local + distant recurrence-free (H) survival in the specified genotypic cohorts. HR, hazard ratio. (**I**) Bar graphs showing co-occurrence and/or mutual exclusivity (Mut Exc) of mutation of each of the four cell cycle checkpoint kinase genes with that of *TP53* across cancer types in three independent datasets: TCGA, MSKCC, and the Pan-China cancer study. Fisher’s exact test determined the *P* values for (A) (with Holm’s adjustment for multiple comparisons), two-tailed Student’s *t* test for (B) to (F), log-rank test for (G) and (H), and Mutual Exclusivity Modules in cancer (MEMo) analysis for (I). #*P* ≤ 0.1, *P ≤ 0.05, **P ≤ 0.01, and **P ≤ 0.001; none, in none of the genes of interest. Supporting data validating activity of compounds used and independent confirmation in other patient tumor datasets are presented in fig. S5.

Unlike mutations in *ATM* or *CHEK2*, mutations in *ATR* alone are uncommon in both primary and metastatic ER^+^/HER2^−^ breast cancer. However, co-incident mutation of *ATR* and *TP53* is 12-fold enriched in metastatic ER^+^/HER2^−^ breast cancer over primary disease (*P* = 0.0002) and twofold enriched over TNBC (*P* = 0.02) (Fig. 7A). In experimental assays, administration of a validated small-molecule ATR inhibitor (fig. S5A) (*34*) significantly promotes motility in wound healing (Fig. 7, B and C) assays, as well as migration (Fig. 7, D and E) and invasion (Fig. 7F) in transwell assays, in *TP53* mutant T47D cells. However, ATR inhibition has no effect on motility or invasion of *TP53* wild-type MCF7 cells (Fig. 7, B to F). Inhibition of ATR in these cells also induces DNA damage as measured by immunofluorescence for markers of double-stranded (γH2AX) and single-stranded (53BP1) DNA damage (fig. S4I). As with CHK2 loss-induced DNA damage, whether this induction of DNA damage is causal to ATR loss-induced metastasis remains unknown.

A p53-dependent role for ATR in metastasis is supported by analyses of patients with ER^+^/HER2^−^ breast cancer: *ATR* mutation alone does not associate with metastasis-free survival, but *ATR*/*TP53* comutation associates significantly with worse metastasis-free survival in two independent datasets {METABRIC [hazard ratios (HR) = 1.89, *P* = 0.004; Fig. 7G] and The Cancer Genome Atlas (TCGA) (fig. S5B)}. This comutation of *ATR/TP53* is also an independent prognostic factor for increased metastatic recurrence in patients with ER^+^/HER2^−^ breast cancer (fig. S5C). Given that *TP53* mutation is a hallmark of ER^−^ breast cancer, we also tested association of *ATR*/*TP53* comutation with metastasis-free survival in ER^−^ disease. We found that *ATR*/*TP53* comutation associates significantly with poor metastasis-free survival in ER^−^ breast cancer in both datasets analyzed (Fig. 7H and fig. S5D). In accordance with these data, while primary ER^+^/HER2^−^ breast cancer is normally highly PR^+^ with decreasing levels in metastatic disease, mutations in *ATR* and *TP53* associate with 1.7-fold higher likelihood of PR negativity even in primary disease relative to wild-type tumors (*P* = 2.7 × 10^−10^) (fig. S5E). Notably, in a pan-cancer analysis of TCGA, Pan-China, and Memorial Sloan Kettering Cancer Center (MSKCC) data (Fig. 7I), *ATR* is the only cell cycle checkpoint kinase that is comutated with *TP53* in all three datasets (cumulative log_2_ odds ratio = 0.968, cumulative *q* < 0.001). Mutation of *ATM* is mutually exclusive with that of *TP53* (cumulative log_2_ odds ratio = −0.55, cumulative *q* < 0.001), while neither *CHEK1* nor *CHEK2* mutation is consistently comutated with *TP53*.

Together, these data support distinctive roles for individual cell cycle checkpoint kinases in metastatic progression of breast cancer, with dysregulation of *ATM* associating with primary ER^+^/HER2^−^ disease, *CHEK2* with metastatic ER^+^/HER2^−^ breast cancer, and *ATR* with metastatic disease that is ER agnostic but reliant on *TP53* mutation.

#### CHK2 dysregulation causally impacts treatment responsiveness in breast cancer

**Fig. 8.**
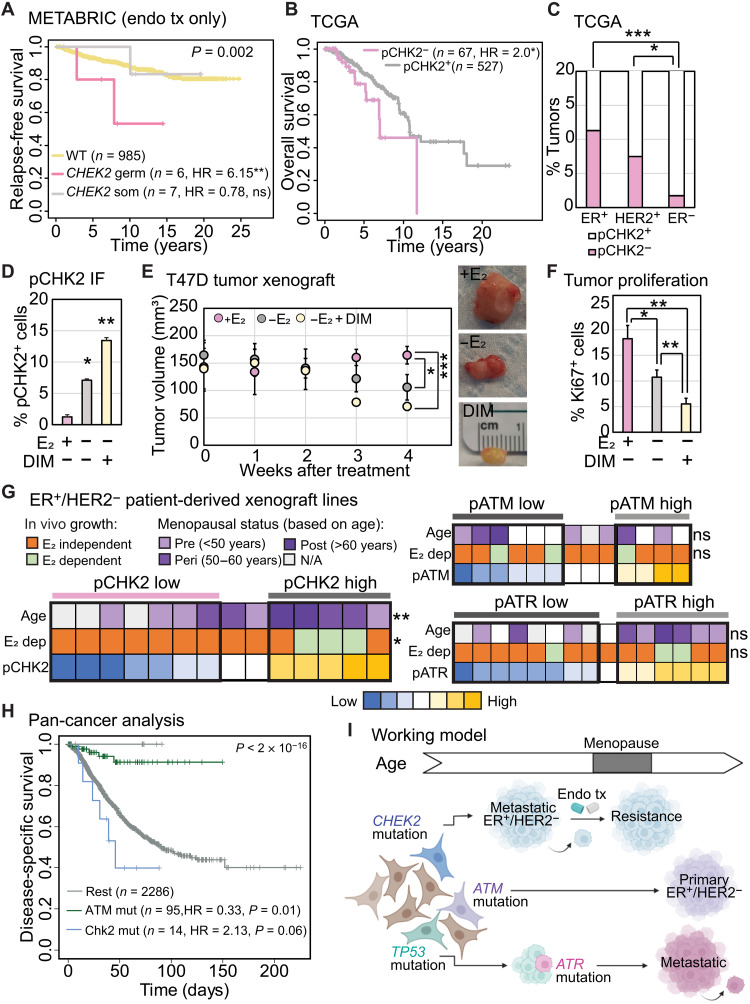
CHK2 activation promotes responsiveness to standard endocrine therapy. (**A**, **B**, and **H**) Kaplan-Meier survival curves measuring specified outcomes in patients with mutated (A) and (H) or down-regulated (B) CHK2 and ATM (H) in breast (A) and (B) and other cancers (H). Log-rank test determined *P* values. (**C**) Stacked column graph representing incidence of CHK2 down-regulation measured by reverse phase proteomics array in ER^+^/HER2^−^ breast cancer samples from TCGA. Fisher’s exact test determined the *P* values. (**D** and **F**) Bar graphs quantifying percentage of pCHK2^+^ and proliferating cells (Ki67^+^) in xenograft tumors derived from ER^+^/HER2^−^ breast cancer cell line, T47D, grown in mice with β-estradiol supplementation in drinking water (Veh), estrogen deprivation (−E_2_), and CHK2 activator, DIM incorporated in chow. (**E**) Dot plot depicting mean tumor volumes in mice xenografted with T47D cells and treated as specified. Three mice per group. Representative images of tumors from specified treatment conditions at harvest. (**G**) Heatmaps indicating protein levels of pCHK2, phospho-ATM (pATM), and phospho-ATR (pATR) across a panel of ER^+^/HER2^−^ breast cancer PDXs. Ability of tumors to grow in the absence of estrogen supplementation and age of diagnosis are represented. Low (mean − SD) and high (mean + SD) phosphorylated/total protein levels were compared for statistically significant differences in age at diagnosis and estrogen-independent growth using Fisher’s exact test. (**I**) Working model depicting the impact of mutations in different cell cycle checkpoint kinase genes on the type of breast cancer a patient may develop and clinical consequences. *P ≤ 0.05, **P ≤ 0.01, and ***P ≤ 0.001. Unless otherwise specified, two-tailed Student’s *t* test determined *P* values and error bars represent SD. Associated data are presented in fig. S6.

We next investigated association of mutations in cell cycle checkpoint kinase genes with response to endocrine treatment using two independent datasets, MSKCC (*48*) and METABRIC (*49*). We found that *CHEK2* mutations in patients with metastatic ER^+^/HER2^−^ breast cancer, whether germ line or somatic, associate with shorter progression-free survival (average of 183 days) on frontline endocrine therapy relative to patients with *CHEK2* wild-type disease (584 days) (MSKCC, *P* = 0.03) (fig. S6A). We found a similar association between incidence of germline *CHEK2* mutations and worse relapse-free survival in patients with primary ER^+^/HER2^−^ breast cancer in METABRIC (HR = 6.15, *P* = 0.01) (Fig. 8A). This association remains significant in a proportional hazards assessment including known prognostic factors of PR status, tumor stage, age at diagnosis, and type of administered endocrine therapy (fig. S6B) and is not detectable in patients with primary ER^+^/HER2^−^ breast cancer who did not receive endocrine therapy (fig. S6C). Notably, we did not observe an association between somatic *CHEK2* mutations and poor outcome in METABRIC. This is likely because there is no significant enrichment for deleteriousness in germline versus somatic *CHEK2* mutations in metastatic breast cancer samples in MSKCC. In contrast, this enrichment is detectable in METABRIC (6 of 6 germline mutations are deleterious versus 3 of 12 somatic mutations, *P* = 0.009 by Fisher’s exact test). We confirmed the association between CHK2 dysregulation and poor outcome for patients with ER^+^/HER2^−^ breast cancer using proteomic data for phosphor-CHK2 (TCGA: HR = 2.0, *P* = 0.02) (Fig. 8B) and validated the observed enrichment for CHK2 dysregulation in ER^+^/HER2^−^ disease relative to either HER2^+^ (1.5-fold) or TNBC (10-fold) at the protein level (*P* = 0.0004; Fig. 8C).

To experimentally test the link between CHK2 dysregulation and endocrine therapy resistance, we administered DIM to mice bearing xenografted tumors from ER^+^/HER2^−^ T47D breast cancer cells. Estrogen deprivation alone activates CHK2 (Fig. 8D) and inhibits tumor growth (*P* = 0.04) (Fig. 8E). However, the addition of DIM activates CHK2 twice as much as estrogen deprivation alone (*P* = 0.02) (Fig. 8D; 11-fold increase over estrogen-supplemented tumors, *P* = 0.007) to further suppress tumor growth (*P* = 0.005) (Fig. 8E) and proliferation (*P* = 0.008) (Fig. 8F). These in vivo data support the hypothesis that CHK2 activation mediates responsiveness to endocrine therapies. As further experimental validation, we conducted a proteo-genomic analysis of phosphor-ATM, phosphor-ATR, and phosphor-CHK2 levels across a panel of ER^+^/HER2^−^ patient-derived xenograft (PDX) lines. We found that low levels of CHK2 phosphorylation predict estrogen-independent growth across PDX lines in vivo (Fig. 8G; seven of seven phosphor-CHK2 low PDX lines are estrogen-independent compared to two of five phosphor-CHK2 high lines, *P* = 0.04). We also found that ER^+^/HER2^−^ breast cancer PDXs with low levels of phospho-CHK2 are more likely to be derived from patients with premenopausal breast cancer (Fig. 8G; median age for phospho-CHK2 low PDXs is 38.5 versus 57 in pCHK2 high lines, *P* = 0.009) in accordance with data from genetically engineered mice presented in Figs. 3 and 4 and the mutational analysis of germline *CHEK2* variants in patient data presented above. Notably, we found no such correlations with either ATM or ATR phosphorylation (Fig. 8G).

To test whether these associations with outcome extend across cancer types, we analyzed whether tumors with somatic mutations in *ATM* or *CHEK2* associate with disease-specific survival in a TCGA pan-cancer dataset (fig. S1B). We observed that mutations in *ATM* associate with improved outcome (HR = 0.52, *P* = 0.0005) in a cancer type–agnostic manner as assessed by Cox proportional hazards analysis (fig. S6D). However, we found no significant (HR = 1.24, *P* = 0.54) association between *CHEK2* mutations and outcome. To test whether the association of *CHEK2* mutation with poor outcome observed in breast cancer extends to other cancer types preferentially in women, we conducted a gender-stratified Kaplan-Meier analysis of disease-specific survival and somatic mutations in *ATM* and *CHEK2* across solid cancers. This analysis identified poorer outcomes for women with somatic mutations in *CHEK2* (HR = 2.13, *P* = 0.06) but not men (HR = 0.78, *P* = 0.73) (Fig. 8H; Cox regression log rank test for *P* values). Similarly, the improved outcome associated with *ATM* mutation across cancer types is pronounced when only considering women (HR = 0.33, *P* = 0.01) (Fig. 8H) and is reduced when the analysis is restricted to men (HR = 0.68, *P* = 0.07). These data suggest that the dichotomous association of mutations in *CHEK2* and *ATM* with survival outcomes is not restricted to breast cancer but extends to other cancer types in a gender-dependent manner.

Overall, these data provide some of the first evidence for divergences in the causal impact of ATM, CHK2, and ATR inactivation on the type of breast cancer a patient develops and on disease progression, i.e., metastatic potential and responsiveness to endocrine therapies (Fig. 8I).

## DISCUSSION

While cell cycle checkpoint kinases are well recognized as tumor suppressors across cancer types, and the efficacy of inhibitors of these proteins (primarily ATR and CHK1) in targeting cancer has been frequently investigated (*50*), we lack systematic understanding of the relative contributions of each of these kinases to cancer initiation and progression. The findings presented in this work constitute the first systematic analysis of the implications of dysregulation of each of these kinases on tumor subtype formation and disease progression that incorporates patient tumor data analysis with experimental validation of associations. Although the primary cancer type investigated in this study is breast cancer, we also present confirmatory analyses in three independent pan-cancer datasets demonstrating the extension of some of these findings across cancer types. Further investigation in individual cancer types other than breast is certainly warranted.

We find that women who are carriers of deleterious, germline variants in *CHEK2* are predisposed to the incidence of premenopausal ER^+^/HER2^−^ breast cancer. Our experimental demonstration in genetically engineered mice suggest that *CHEK2* mutation–induced tumorigenesis requires the premenopausal mammary environment, which is an important initial evidence of a complex interplay between hormones, aging, and cell cycle checkpoint signaling. This finding is supported by previous reports of a 4% incidence rate for CHEK2*1100delC mutation in patients with premenopausal breast cancer (*51*) versus a 0.7% incidence rate in patients with postmenopausal breast cancer (*52*) in a population background of ~0.2% (calculated from ClinVar). These data raise the hypothesis that different cell cycle checkpoint kinases may be preferentially involved in promoting cancer in young people, which requires in-depth investigation. These results also raise the translationally important question (*53*) of whether cancer screening in *CHEK2* germline variant careers should be modulated on the basis of age and menopausal status to prevent overdiagnosis and overtreatment.

In addition, we find that breast cancer patients with either somatic or germline mutations in *CHEK2* are more likely to be diagnosed with metastatic than primary ER^+^/HER2^−^ breast cancer. These data are supported by previous studies showing that (i) mammary tumors growing in CHEK2*1100delC mice crossed with the MMTV-Ron kinase mammary tumor–susceptible mouse line are highly metastatic (*42*); (ii) there is increased incidence of germline *CHEK2* mutations in metastatic breast cancer relative to primary disease (*54*); and (iii) that *CHEK2* mutation carriers have worse recurrence-free breast cancer survival outcomes (*55*). To our knowledge, results presented here constitute the first experimental validation of a direct role for CHK2 inhibition in promoting metastatic phenotypes in ER^+^/HER2^−^ breast cancer cells, although studies from other groups indicate a role for CHK2 activation in epithelial mesenchymal transition (*56*, *57*), often a precursor to metastasis. Mechanisms that underlie the causal link between CHK2 inactivation and metastatic phenotypes warrant further study with additional comprehensive in vivo experiments using orthogonal experimental model systems such as intraductal injections.

We also find that breast cancer patients with *CHEK2* mutations appear resistant to endocrine monotherapy, which targets the ER signaling pathway, despite their cancer being highly PR^+^. High PR positivity is considered a sign of dependence on ER signaling. In cell line and PDX tumor growth studies, we demonstrate that CHK2 dysregulation alters estrogen dependence in vivo. We also observed increase in apoptotic cells in CHEK2*1100delC homozygous mutants, consistent with previous reports, likely due to the increased genomic instability caused by CHK2 loss (*40*, *42*). We identify higher levels of markers of double-stranded and single-stranded DNA breaks in preneoplastic lesions of homozygous *CHEK2* mutant mice and in cell lines after administration of CHK2 inhibitors, indicating impairment of the DNA damage response. These data align with previous reports suggesting that defective DNA damage response with concomitant inability to activate CHK2 can cause endocrine treatment resistance (*26*). In this context, the efficacy of CHK2 activators in inhibiting tumor growth and metastasis in vivo also suggests alternate avenues for next generation cell cycle–based cancer therapeutics. Because ATM and CHK2 inhibitors are largely ineffective in cancer clinical trials (*58*), development of activators of these kinases might afford more complete cell cycle control especially in conjunction with cyclin-dependent kinase 4/6 (CDK4/6) inhibitors that have shown efficacy in the clinic (*59*). Overall, these findings provide a comprehensive portrait of the distinctive impact of CHK2 dysregulation on the evolution of premenopausal, metastatic, highly PR^+^, ER^+^/HER2^−^ breast cancer that is likely to resist standard endocrine monotherapies.

Conversely, we find that *ATM* mutations enrich for incidence of primary ER^+^/HER2^−^ breast cancer that is preferentially PR^−^. PR negativity in patients with ER^+^/HER2^−^ breast cancer associates with more aggressive disease that may be less responsive to endocrine therapies (*25*, *60*). However, we did not find associations between *ATM* mutations and poor patient outcome in this study. While ATM and CHK2 are often paired together in canonical cell cycle regulation and DNA damage response (*29*, *61*, *62*), the results of our study suggest that they have distinct cellular functions that underlie their individual impact on breast cancer presentation and progression. It is also possible that redundancies between ATM, ATR, and DNA-PKcs in phosphorylating and therefore activating CHK2 (*9*, *63*) may be responsible for the later onset clinical presentation of breast cancer in *ATM* mutation carriers. Alternately, previous reports of distinct roles for these two kinases in G_1_ checkpoint regulation may constitute the mechanism underlying their differential impact on cancer phenotypes (*64*). Larger datasets and more mechanistic studies are required to understand how ATM dysregulation affects disease progression and patient outcome.

Last, as a previously unidentified observation, we uncover a role for comutation of *ATR* and *TP53* in breast cancer metastasis. The ability of p53 mutational context to modulate the oncogenic/tumor suppressive ability of many driver genes [e.g., Myc (*65*) and TLR4 (*66*)] is well recognized. In the same vein, both in patient tumor data and in experimental analyses, we find that loss of ATR only affects tumor phenotypes and biology in the context of dysregulated p53. Further, the association of this comutation with metastasis-free survival appears significantly stronger than that of mutation of either gene alone. This dichotomy may explain why previous epidemiological studies considering tumors of both wild-type and mutant p53 status failed to find associations between ATR dysregulation and breast cancer outcome. This comutation of *ATR* and *TP53* appears consistent across many cancer types in pan-cancer analyses presented here. Therefore, these results are translationally critical in informing the use of ATR inhibitors in clinic to selectively target *TP53* wild-type cancers. It is also important to point out that this comutation effect is only observed for *ATR* and not for the other cell cycle checkpoint kinases.

Overall, this systematic analysis of the association of individual cell cycle checkpoint kinase genes with clinically relevant tumor characteristics and breast cancer patient outcome suggests that knowledge of the mode of cell cycle dysregulation during tumorigenesis can be effectively leveraged to improve characterization of cancer subtypes. One caveat in this regard is that the four cell cycle checkpoint kinases described here are not the only modulators of the cell cycle or of DNA damage response; other important kinases such as DNA-PKCs (*9*) have not been considered in this analysis but undoubtedly warrant further investigation. While breast cancer is an excellent cancer type for these proof-of-concept analyses and our initial exploratory analyses suggest that these findings are likely to be relevant across other cancer types as well, there is a need for nuanced investigation into how the mode of cell cycle dysregulation affects the evolution and progression of other cancer types within age- and gender-dependent contexts.

With successful clinical implementation of CDK inhibitors for certain types of cancer, there is renewed interest in understanding how different cyclin and CDK dependencies in cancer cells can guide therapeutic decisions (*67*). As upstream regulators of these cyclins and CDKs, ATM, ATR, CHK1, and CHK2 are among the most common cell cycle dysregulation events that promote cancer susceptibility across cancer types (*68*). The results of this study, by demonstrating clear divergences in the impact of dysregulation of ATM, ATR, and CHK2 on important tumor characteristics, shed new insight into how early decisions to turn off cell cycle regulation can direct the course of ensuing disease. Simple inhibition of each kinase as tested in the clinic (*69*, *70*) may, therefore, not be an optimal solution. Further evidence that early loss of specific cell cycle checkpoint kinases essentially serves as a decision point for evolution of cancers with specific prognostic and progressive tendencies may argue for an improved system of cancer classification based on the mode of cell cycle checkpoint inactivation to guide selection of therapeutics. Development of these prognostic and predictive stratifiers could provide new strategies to match CDK inhibitors or next-generation cell cycle checkpoint activators to the individual cell cycle dependencies of each patient’s tumor.

## METHODS

### Datasets

Six datasets with mutational data from patients with primary and metastatic breast cancer were combined for initial analyses (see Fig. 1 and fig. S1). The results included here use data from The Metastatic Breast Cancer Project (www.mbcproject.org/), part of Count Me In (https://joincountmein.org/). Analyses regarding patient outcomes were conducted in two independent datasets: MSKCC (*71*) and METABRIC. Three pan-cancer datasets, TCGA/International Cancer Genome Consortium (ICGC), MSKCC, and the Pan-China (*72*) dataset were also analyzed. Details of each dataset are presented in Supplementary Methods.

### Mutational analysis

All protein changing mutations in *TP53*, *ESR1*, *ATM*, *CHEK2*, *ATR*, and *CHEK1* genes were included irrespective of category (i.e., missense, nonsense, etc.) or predicted pathogenicity. Protein changing mutations in *TP53* and *ESR1* genes served as controls as they are known drivers of TNBC and ER^+^/HER2^−^ breast cancer, respectively. Mutational frequency was calculated on the basis of the total number of mutations divided by patient count.

### Tumor characteristics

Tumor PR status and age of diagnosis served as categorical variables to determine associations between incidence of mutations and patient/tumor characteristics. Fisher’s exact test was used to determine *P* values by comparing different categories such as ER^+^/HER2^−^ versus TNBC or germline versus somatic status for PR positivity, while a two-tailed Student’s *t* test was used for continuous age differences.

### Cell lines

MCF7 and T47D ER^+^/HER2^−^ breast cancer cell lines are used throughout for in vitro experiments. Both lines are validated annually for authenticity. MCF7 is wild-type for *TP53*, while T47D is *TP53* mutant (L194F) and a commonly used model for loss of *TP53* function. Both lines are wild-type for all components of the mismatch repair pathway.

### Genetically engineered mice

The mice in the 5-month premenopausal experiment were from either strain C57BL/6N [line: Atm1BrdChek2tm1a(EUCOMM)Hmgu/JMmucd], which were received from Mutant Mouse Resource and Research Center (MMRRC) (catalog no. 047089-UCD) or strain 129/Sv*BlackSwiss*FVB/N (line: Chek2tm1Pjs/Mmnc), which were also received from MMRRC (catalog no. 01411-UNC). The mice in the 18-month postmenopausal and VCD-induced menopause experiment were only from strain 129/Sv*BlackSwiss*FVB/N. The 129/Sv*BlackSwiss*FVB/N lines were backcrossed to FVB mice for six generations to stabilize the strain background. For all mice, genotyping was done when mice were 4 to 6 weeks old. The C57BL/6N and 129/Sv*BlackSwiss*FVB/N mice were genotyped according to MMRRC protocol with bands expected around 500 base pairs. MMTV-Ron/CHEK2*1100delC mice were derived as described by Meyer *et al.* (*42*).

### In vivo experiments

For the 5-month and 18-month experiments, mice were genotyped at 4 to 6 weeks and housed in random groups between 6 and 8 weeks old and then harvested at 5 and 18 months, respectively. Mice were palpated once monthly until tumors were palpable. Once a tumor was palpable, palpations were done weekly. None of the 5-month mice developed palpable tumors. Of the 59 mice in the 18-month experiment, 23 mice (11 +/+, 5 +/m, and 7 m/m) were found dead with cause of death unknown. Details of the VCD-induced menopause experiment [as per published protocol (*73*)] are in Supplementary Methods. All mammary fat pads were harvested from the 5-month premenopausal, 5-month induced menopause, and 18-month postmenopausal experiments. The left #4 and left #2/3 were fixed in 4% paraformaldehyde and paraffin embedded, the right #2/3 was snap frozen, and right #4 was whole-mounted and stained with neutral red as previously described (*55*). Mice for the xenograft experiment were 6- to 8-week-old Non-Obese Diabetic/Severe Combined Immunodeficieny (NOD/SCID) mice (from Sanford Burnham Prebys). Mice were injected in the left #4 mammary gland with T47D cells suspended in Matrigel (Corning catalog no. 356234) and randomized into three treatment groups (+E_2_, −E_2_, and −E_2_ + DIM) when tumors reached 100 mm in diameter. Similarly, the tail vein injection experiment was done using 6- to 8-week- old NOD/SCID mice. T47D cells were suspended in phosphate-buffered saline and then injected into the tail vein. Mice were randomized into three treatment groups (Veh, Fulv, and Fulv + DIM). Mice were injected with luciferin and imaged with an IVIS imager to monitor metastasis. After 6 weeks, mice were harvested as previously described (*74*). Estradiol was supplemented into sterile deionized water at a concentration of 8 μg/ml, and DIM (MedChemExpress) was given in diet form (Research Diets).

### Immunostaining

Immunofluorescence was performed based on manufacturer’s instructions and as per previously published protocols (*74*). Antibodies used include phosphor-Chk2 (Cell Signaling Technology, catalog no. 2197S), ER (EMD Millipore, catalog no. 04820MI), 53BP1 (Novus Biologicals, catalog no. NB100-304), gH2AX (Cell Signaling Technology, catalog no. 9718), and Ki67 (Abcam, catalog no. ab16667). Immunohistochemistry was performed on the basis of manufacturer’s instructions. Sections were first deparaffinized, then endogenous peroxidases were quenched using 3% H_2_O_2_, and antigen retrieval was done using 1× citric acid buffer. The blocking buffer used was 2% goat serum. Antibodies used were ER, Ki67 (Abcam, catalog no. ab16667), cleaved caspase-3 (Cell Signaling Technology, catalog no. 9664), and PR (Thermo Fisher Scientific, catalog no. MA512658). Primary antibodies were left overnight in 4° and followed by anti-rabbit secondary (Vector Laboratories, catalog no. BA-1000) or anti-mouse secondary (Vector Laboratories, catalog no. MKB-2225). Next, sections were incubated in avidin-biotin complex solution (Vector Laboratories, catalog no. PK-6100), stained with peroxidase substrate (Vector Laboratories, catalog no. SK-4800), and counterstained in hematoxylin. Images were captured on an Echo Revolve microscope.

### Migration and invasion assays

Wound healing and transwell migration assays were performed to assess metastatic potential. For wound healing assays, 2.5 × 10^5^ T47D cells were plated in six-well plates and incubated for 24 hours. Using a 20-μl pipette tip, a scratch was made in the center of the plate and pictures were taken at 0, 24, and 48 hours. Fresh media with 100 nM ATR inhibitor (Selleckchem, catalog no. S8007), CHK2 inhibitor (Sigma-Aldrich, catalog no. C3742), or 1 mM CHK2 activator, DIM (Santa Cruz Biotechnology, catalog no. sc-204624B) were added and incubated for 48 hours. Pictures were taken at 48 hours to quantify wound healing. For transwell migration and invasion assays, 2.0 × 10^5^ cells in 200 μl of media [no fetal bovine serum (FBS) + ATR or CHK2 inhibitors] were added in transwell inserts (Falcon, catalog no. 353182). For invasion assay, inserts were coated with Matrigel (Corning, catalog no. 356234) in 1:3 dilution with media without FBS. Inserts were placed on 12-well plate with 750 μl of cell culture media. Fixation and staining were carried out after overnight incubation. Transwells were placed in fixative (Thermo Fisher Scientific, catalog no. 22-122911) followed by 5 min staining in solution (Thermo Fisher Scientific, catalog no. 22–122911). DIM transwell experiments were conducted in media with charcoal-stripped serum and estradiol supplementation, while all inhibitor experiments were conducted in media with full serum. Inserts were dried, and pictures were taken using Echo microscope.

### Biomatrix assays

The T47D breast cancer cell line was cultured on a hanging drop array to form three-dimensional tumor spheroids over the course of 4 days (*75*, *76*). On day 3, the spheroids were either treated with 10 μM DIM inhibitor or fed with fresh media for control. After compact spheroid formation was confirmed (day 4), the cells were seeded onto a decellularized lung biomatrix scaffold to establish engineered breast cancer lung metastasis (BCLM) and multiphoton imaging per previously established protocols (*77*). The cells were allowed 2 hours for matrix attachment before undergoing 3-(4,5-dimethylthiazol-2-yl)-5-(3-carboxymethoxyphenyl)-2-(4-sulfophenyl)-2H-tetrazolium (MTS) metabolic assay or being submerged in culture medium (+/− DIM) for continued growth. Following 4 days of culture, engineered BCLM was harvested to measure metabolic activity via absorbance-based MTS assay.

### Western blotting

Western blotting was conducted as previously described. Cells were exposed to 48 hours of ATR or CHK2 inhibitor/activator treatments. All antibodies were diluted in 1× Tris buffered saline with Tween (TBST) in 1:1000 dilution and incubated overnight at 4°C. Antibodies used were phospho-ATR (Cell Signaling Technology, catalog no. 2853), ATR (Cell Signaling Technology, catalog no. 2790), phospho-Chk2 (Cell Signaling Technology, catalog no. 2197), Chk2 (Cell Signaling Technology, catalog no. 2662), phospho-ATM (Abcam, catalog no. ab36810), and glyceraldehyde phosphate dehydrogenase (Santa Cruz Biotechnology, catalog no. sc-47724).

### Survival and disease progression analyses

Outcome measures used were relapse-free, metastasis-free, and local/distant recurrence-free survival. Only samples with survival metadata were included. Cox proportional hazards calculated HRs and *P* values. Tumor stage, PR status, age at diagnosis, and classes of endocrine therapy were included in multivariate analyses.

### Statistical analyses

Missing data were imputed with “NA” from mutation and survival data analysis. Samples classifying for more than one category were treated as separate set for statistical comparisons. Independent sample Student’s *t* test was used for age comparisons, and Fisher’s exact test was used for categorical data. Log-rank test calculated *P* values for survival analyses. For analyses where multiple hypotheses were tested, Holm’s adjustment for multiple comparisons was used.

### Rigor and reproducibility

Sample size for experiments was determined on the basis of prior experience conducting similar experiments. Researchers were blinded to groups during quantification of results. For all in vitro experiments, at least three biological replicates were included in each analytical unit and each experiment was conducted at least two times at different times and with different cell line passages. All in vivo experiments were conducted in accordance with the regulatory oversight of, and approved by, the Sanford Burnham Prebys IACUC. Overall, no data points were excluded from analysis unless otherwise explicitly stated in the text of the manuscript. Exclusion and inclusion criteria for the bioinformatics analyses were established before conducting the study.

## Supplementary Materials

### This PDF file includes:

Figs. S1 to S6 Supplementary Methods References

## Notes

View/request a protocol for this paper from *Bio-protocol*.
