# Supplementary material for: Molecular portraits of cell cycle checkpoint kinases in cancer evolution, progression, and treatment responsiveness
Source: Sci Adv. 2023 Jun 30;9(26):eadf2860. doi: 10.1126/sciadv.adf2860 (PMC10313178; doi:10.1126/sciadv.adf2860)
Supplement: Supplementary file 1 — Figs. S1 to S6 Supplementary Methods References [file sciadv.adf2860_sm.pdf]

Science Advances
AAAS

Supplementary Materials for

Molecular portraits of cell cycle checkpoint kinases in cancer evolution, progression, and treatment responsiveness

Elena Oropeza *et al.*

Corresponding author: Matthew N. Bainbridge, mbainbridge@rcigm.edu;
Svasti Haricharan, sharicharan@sbpdiscovery.org

Sci. Adv. **9**, eadf2860 (2023)
DOI: 10.1126/sciadv.adf2860

This PDF file includes:

Figs. S1 to S6
Supplementary Methods
References

Supplementary Figures and Legends

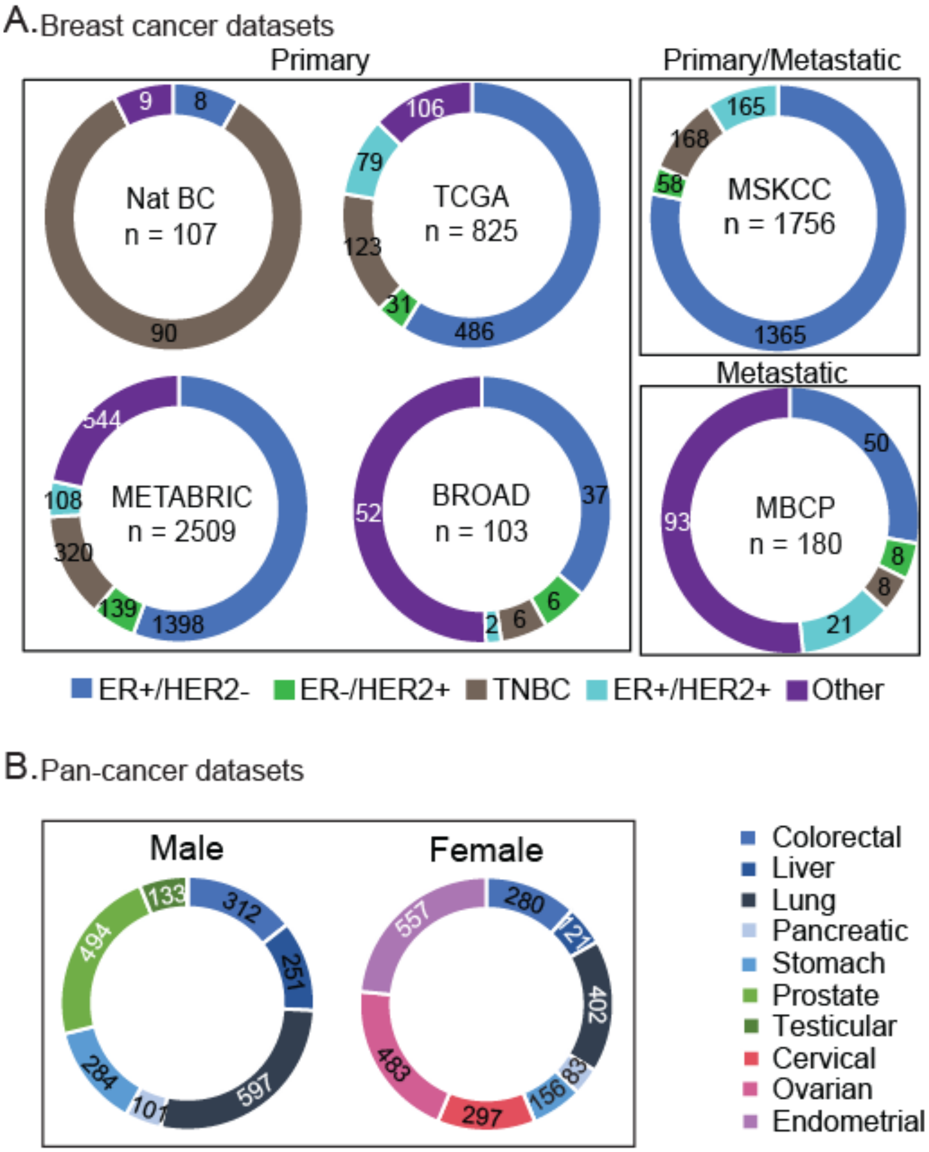

Supplementary Figure 1: Description of samples by patient datasets. Donut plots demonstrate the proportion of tumors from each of the six datasets used divided based on hormone receptor (HR) status and HER2 positivity (**A**) and the proportion of tumors from each cancer type in the TCGA pan-cancer unique dataset from cBioPortal (**B**). TNBC, Triple negative breast cancer. Other indicates samples where one or more of the receptor status variables had missing information. Nat BC, Nature British Columbia; TCGA, The Cancer Genome Atlas; MSKCC, Memorial Sloan Kettering Cancer Center; METABRIC, Molecular Taxonomy of Breast Cancer International Consortium; BROAD, Broad Institute; MBCP, Metastatic Breast Cancer Project. Supports analyses presented in **Figure 1**.

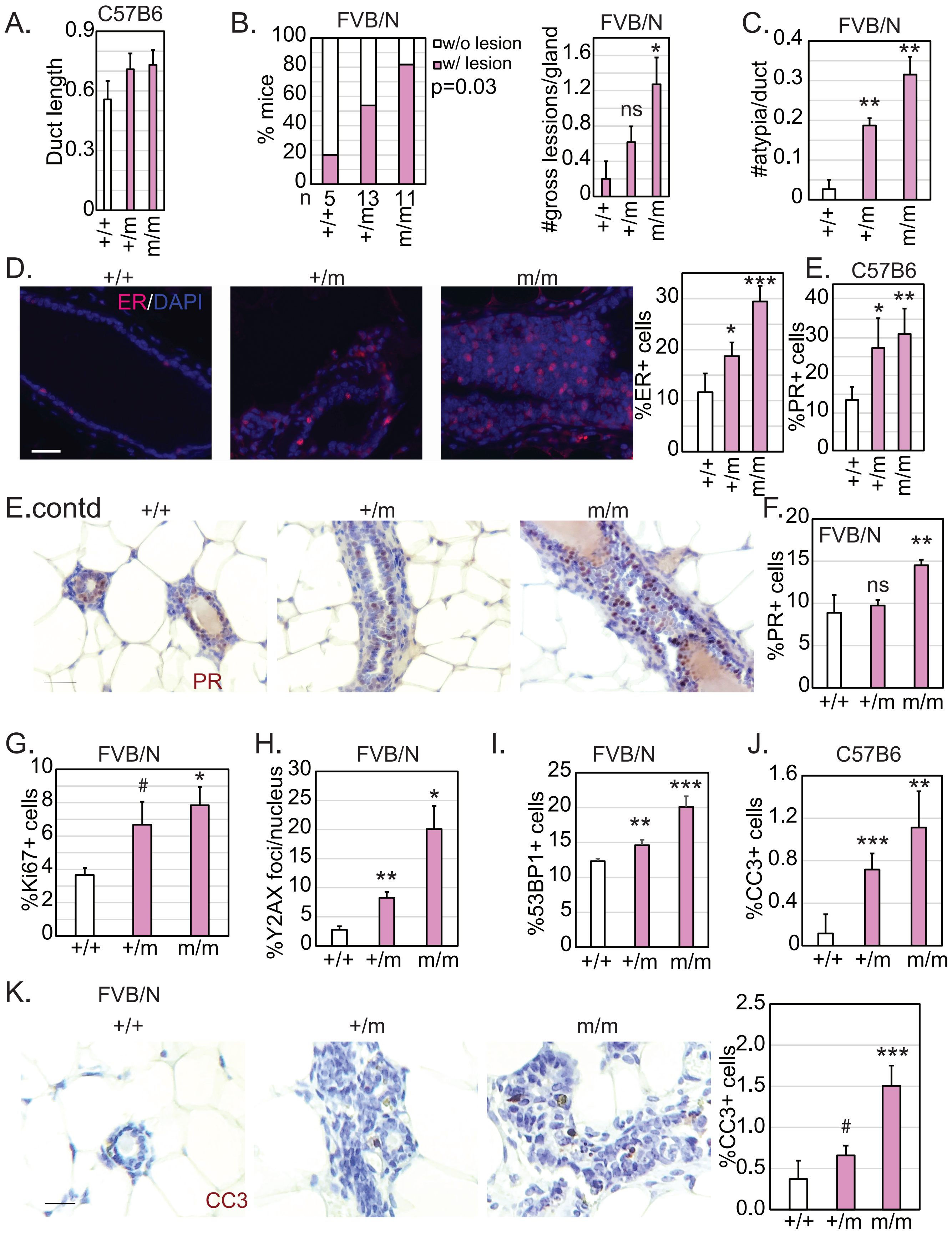
A.
C57B6
Duct length
+/+
+/m
m/m
B.
FVB/N
% mice
w/o lesion
w/ lesion
p=0.03
n
5
13
11
#gross lesions/gland
ns
C.
FVB/N
#atypia/duct
D.
+/+
+/m
m/m
ER/DAPI
%ER+ cells
E.
C57B6
%PR+ cells
E.contd
PR
F.
FVB/N
%PR+ cells
G.
FVB/N
%Ki67+ cells
H.
FVB/N
%γ2AX foci/nucleus
I.
FVB/N
%53BP1+ cells
J.
C57B6
%CC3+ cells
K.
FVB/N
CC3
%CC3+ cells

Supplementary Figure 2. ***CHEK2*** **mutation induces the formation of highly proliferative mammary lesions in genetically engineered mice (FVB background).** **(A)** Bar graph representing quantification of total ductal length (arbitrary units, AU) in mammary glands from female *CHEK2**1100delC mice in the C57/B6 strain background. **(B)** Stacked column graph quantifying incidence (w/lesion, with lesion; w/o lesion, without lesion) and bar graph representing number of gross (macroscopic) mammary lesions. Statistical differences in incidence of lesions tested using Fisher's Exact test, and in number of lesions using Student's T-test. **(C)** Bar graph quantification of the number of microscopic atypia using hematoxylin and eosin staining. **(D-F)** Representative images for ER immunofluorescence **(D)** and PR immunohistochemistry **(E)** alongside bar graphs describing quantification of the same in the indicated mouse strains. **(G-K)** Bar graphs representing percent proliferating cells using immunofluorescence for Ki67 **(G),** percent cells with DNA damage foci using immunofluorescence for gH2AX **(H)** and 53BP1 **(I)**, and percent apoptotic cells with immunohistochemistry for cleaved caspase 3 **(J-K)** in indicated mouse strains. Scale bars = 20µm. Student's T-test derived p-values unless otherwise indicated. For all panels, wildtype (+/+), heterozygous (+/m) and homozygous (m/m) *CHEK2**1100delC mice were harvested at 20 weeks (5 months) of age. Error bars in all bar graphs represent standard deviation. ER, estrogen receptor; PR, progesterone receptor; CC3, cleaved caspase 3; ns, not significant; $p \leq 0.1^{\#}$, $p \leq 0.05^{*}$, $p \leq 0.01^{**}$, $p \leq 0.001^{***}$. Supports data presented in **Figure 3**.

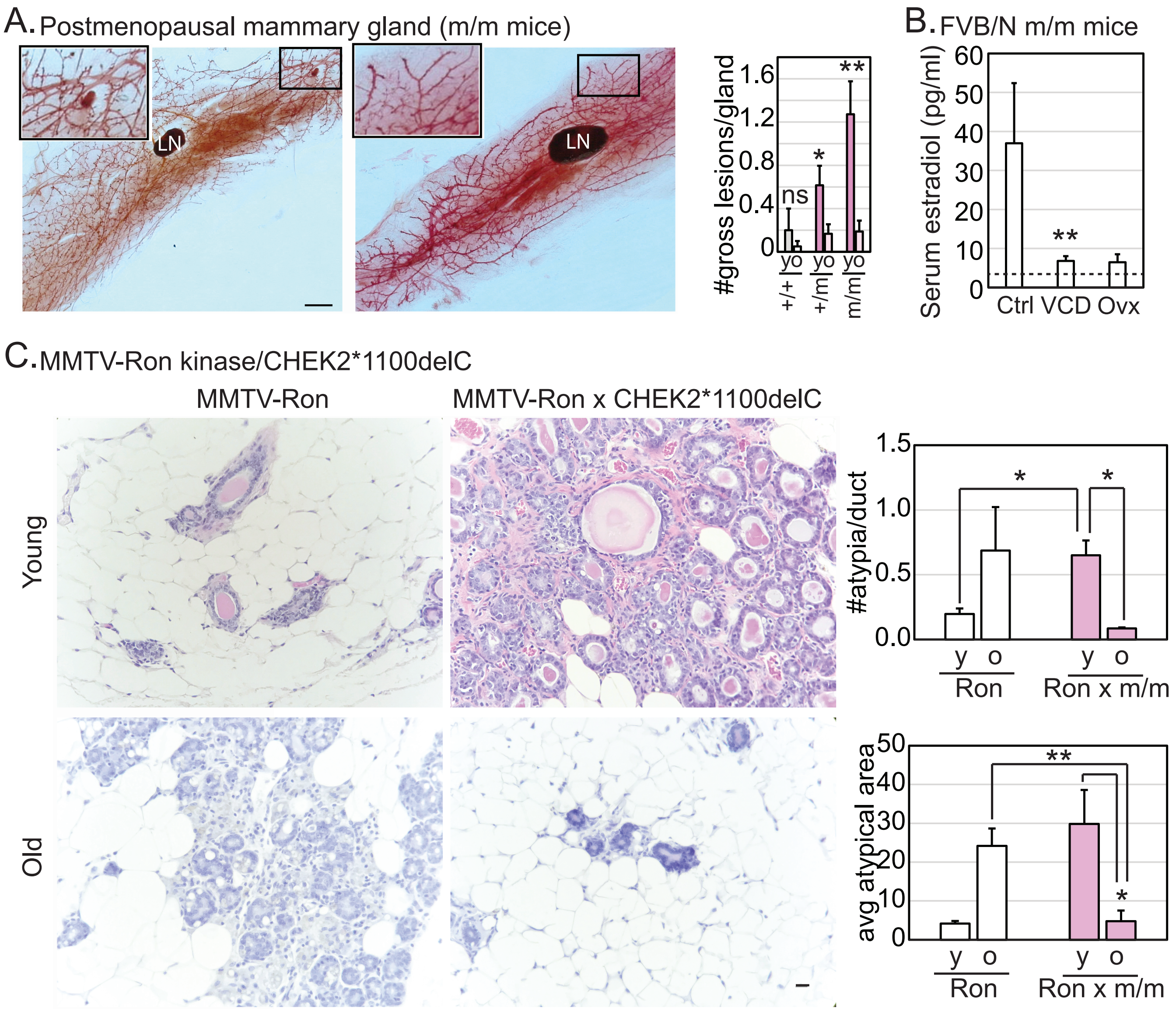
A. Postmenopausal mammary gland (m/m mice)
LN
LN
#gross lesions/gland
1.6
1.2
0.8
0.4
0
ns
*
**
yo
yo
yo
+/+
+/m
m/m
B. FVB/N m/m mice
Serum estradiol (pg/ml)
60
50
40
30
20
10
0
**
Ctrl
VCD
Ovx
C. MMTV-Ron kinase/CHEK2*1100delC
MMTV-Ron
MMTV-Ron x CHEK2*1100delC
Young
Old
#atypia/duct
1.5
1.0
0.5
0.0
*
*
y
o
y
o
Ron
Ron x m/m
avg atypical area
50
40
30
20
10
0
**
*
y
o
y
o
Ron
Ron x m/m

Supplementary Figure 3. ***CHEK2*** **mutation induces formation of highly proliferative mammary lesions in young mice.** (**A**) Representative images and accompanying bar graph quantifying number of macroscopic (gross) lesions per mammary gland in female wildtype (+/+), heterozygous (+/m) and homozygous (m/m) *CHEK2**1100delC mice harvested at 18 months of age. Scale bars = 20µm. Inset magnification 2.5x. (**B**) Bar graph depicting serum estradiol levels in 5 month old mice administered the indicated treatments (Ctrl, control; Ovx, ovariectomy). Dotted line represents serum estradiol levels in postmenopausal (18 month old) female *CHEK2* wildtype FVB mice. **(C)** Representative images and accompanying bar graph quantification of the number and area of microscopic atypia using hematoxylin and eosin (H/E) staining in mammary glands from mice of specified ages and genotypes (young=10-12 months; old=15-18 months). Scale bars = 50µm. Two-tailed Student's T-test determined p-values. Whiskers in all bar graphs represent standard deviation. ns, not significant; $p \leq 0.1^{\#}$, p≤0.05*, p≤0.01**, p≤0.001***. LN, lymph node; ns, not significant; $p \leq 0.1^{\#}$, p≤0.05*, p≤0.01**, p≤0.001***. Supports data presented in **Figure 4**.

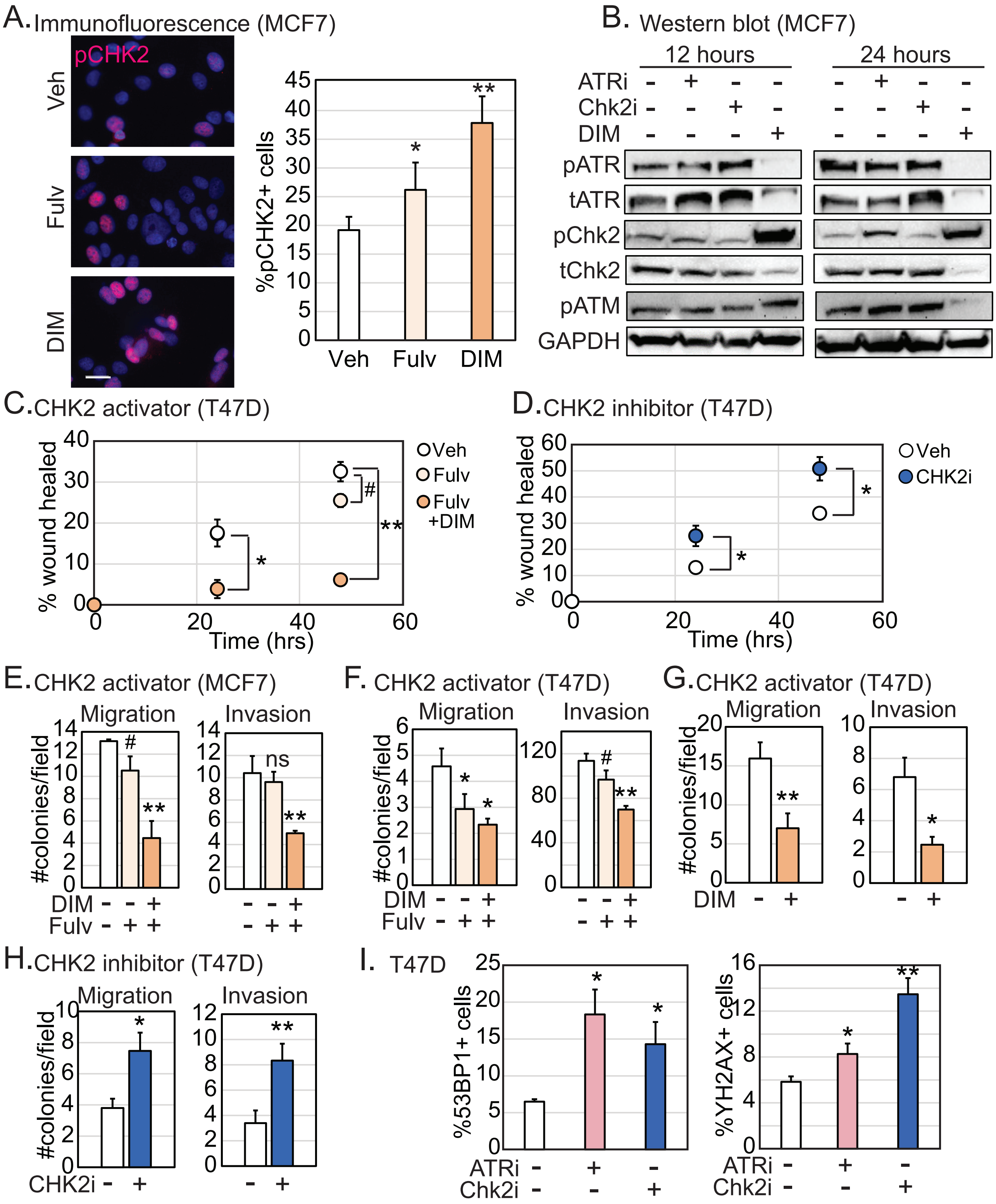
A. Immunofluorescence (MCF7)
pCHK2
Veh
Fulv
DIM
%pCHK2+ cells
B. Western blot (MCF7)
12 hours
24 hours
ATRi
Chk2i
DIM
pATR
tATR
pChk2
tChk2
pATM
GAPDH
C. CHK2 activator (T47D)
% wound healed
Time (hrs)
Veh
Fulv
Fulv +DIM
D. CHK2 inhibitor (T47D)
Veh
CHK2i
E. CHK2 activator (MCF7)
Migration
Invasion
#colonies/field
F. CHK2 activator (T47D)
G. CHK2 activator (T47D)
H. CHK2 inhibitor (T47D)
I. T47D
%53BP1+ cells
%YH2AX+ cells
ATRi
Chk2i

Supplementary Figure 4: CHK2 dysregulation modulates metastatic phenotypes in ER+/HER2- breast cancer cells. (A) Bar graphs representing quantification of nuclear phospho-CHK2 positivity in MCF7 cells treated with vehicle (Veh), Fulvestrant (Fulv, 100nM) or DIM (10µM) for 36 hours, along with representative photomicrographs. Scales bars represent 20µm. **(B)** Western blot demonstrating effect of ATR and CHK2 inhibitors on ATM, ATR and CHK2 phosphorylation. **(C-D)** Dot plots representing quantification of area of scratch at 0, 24 and 48 hours after specified treatments with error bars depicting standard deviation. **(E-H)** Bar graphs representing quantification of transwell migration and invasion assays at 48 hours after specified treatments. **(I)** Bar graphs quantifying the number of nuclei with DNA damage foci through immunofluorescence for 53BP1 and γH2AX in T47D cells *in vitro*. Error bars represent standard deviation. Two-tailed Student's T-test derived p-values. All DIM experiments were conducted in media containing charcoal stripped serum supplemented with β-estradiol while all inhibitor experiments were conducted using media with full serum. not significant, ns; $p \leq 0.1^{\#}$; $p \leq 0.05$*; $p \leq 0.01$**; $p \leq 0.001$***. Supports data presented in **Figures 5-6**.

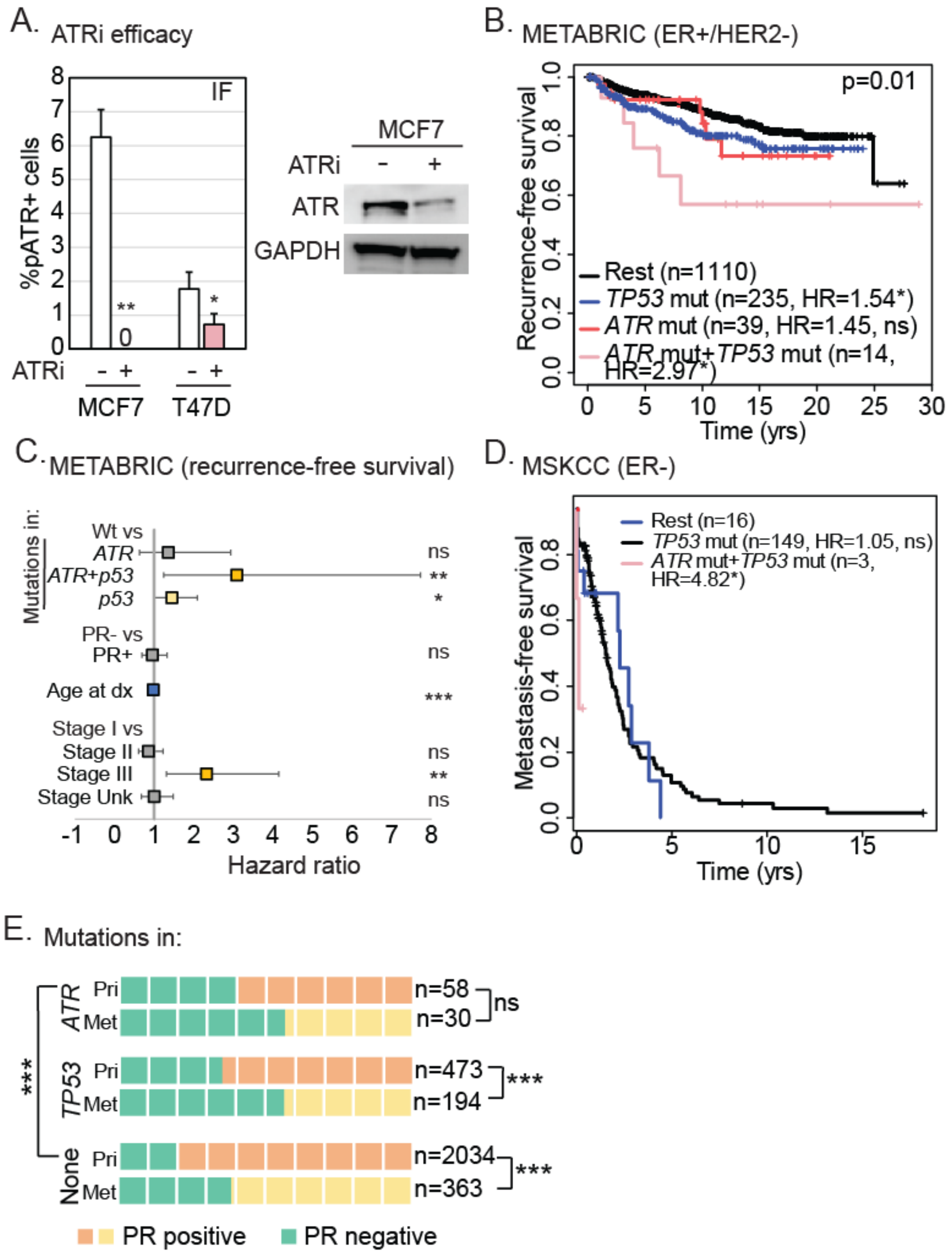
A. ATRi efficacy
IF
%pATR+ cells
ATRi
MCF7
T47D
ATR
GAPDH
B. METABRIC (ER+/HER2-)
p=0.01
Recurrence-free survival
Rest (n=1110)
TP53 mut (n=235, HR=1.54*)
ATR mut (n=39, HR=1.45, ns)
ATR mut+TP53 mut (n=14, HR=2.97*)
Time (yrs)
C. METABRIC (recurrence-free survival)
Mutations in:
Wt vs
ATR
ATR+p53
p53
PR- vs
PR+
Age at dx
Stage I vs
Stage II
Stage III
Stage Unk
Hazard ratio
D. MSKCC (ER-)
Rest (n=16)
TP53 mut (n=149, HR=1.05, ns)
ATR mut+TP53 mut (n=3, HR=4.82*)
Metastasis-free survival
Time (yrs)
E. Mutations in:
ATR
TP53
None
Pri
Met
n=58
n=30
n=473
n=194
n=2034
n=363
PR positive
PR negative

Supplementary Figure 5. Association of ATR dysregulation with metastasis. (A) Bar graph with quantification of pATR positivity assayed by immunofluorescence (IF) of MCF7 and T47D cells, and Western blot demonstrating efficacy of ATR inhibitors (100nM for 48 hours) on inhibiting ATR phosphorylation in MCF7 cells. GAPDH used as loading control. **(B&D)** Kaplan-Meier survival curves measuring the specified outcomes in tumors with mutations in indicated genes. Log rank test determined p-values. **(C)** Forest plots depicting the hazard ratio of indicated survival parameters in a Cox proportional hazards analysis with standard prognostic factors. Boxes indicate the hazard ratio and error bars indicate the 95% confidence intervals. A yellow box indicates a statistically significantly higher hazard ratio, and a blue box indicates a statistically significant lower hazard ratio relative to reference. (**E**) Waffle plot showing proportion of progesterone receptor (PR) positive and negative tumors in cohorts with mutations in indicated genes. Supports data presented in **Figure 7**. ATRi, ATR inhibitor; ns, not significant; HR, hazard ratios; yrs, years; Wt, wildtype; ER, estrogen receptor; Unk, unknown.*, $p<0.05$; **, $p<0.01$; ***, $p<0.001$.

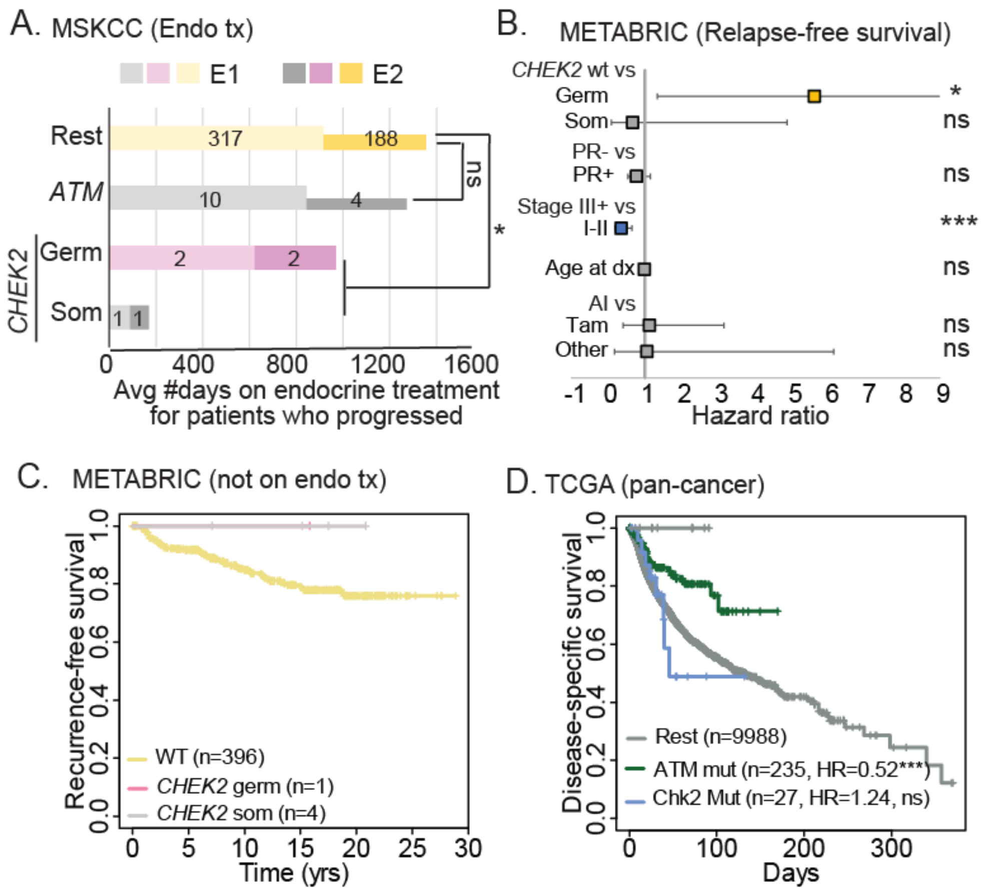
A. MSKCC (Endo tx)
E1
E2
Rest
ATM
CHEK2
Germ
Som
317
188
10
4
2
2
1
1
ns
*
0
400
800
1200
1600
Avg #days on endocrine treatment
for patients who progressed
B. METABRIC (Relapse-free survival)
CHEK2 wt vs
Germ
Som
PR- vs
PR+
Stage III+ vs
I-II
Age at dx
AI vs
Tam
Other
*
ns
ns
***
ns
ns
ns
-1 0 1 2 3 4 5 6 7 8 9
Hazard ratio
C. METABRIC (not on endo tx)
Recurrence-free survival
0.0 0.2 0.4 0.6 0.8 1.0
WT (n=396)
CHEK2 germ (n=1)
CHEK2 som (n=4)
0 5 10 15 20 25 30
Time (yrs)
D. TCGA (pan-cancer)
Disease-specific survival
0.0 0.2 0.4 0.6 0.8 1.0
Rest (n=9988)
ATM mut (n=235, HR=0.52***)
Chk2 Mut (n=27, HR=1.24, ns)
0 100 200 300
Days

Supplementary Figure 6. CHK2 loss associates with worse patient outcome. **(A)** Stacked column graph depicting the length of time for which metastatic ER+/HER2- breast cancer patients stayed on their first (E1) and second (E2) lines of endocrine treatment before progressing categorized based on the presence of somatic (Som) or germline (Germ) mutations in *ATM* or *CHEK2*. Fisher's Exact test determined p-values. **(B)** Forest plots depicting the hazard ratio of indicated survival parameters in a Cox proportional hazards analysis with standard prognostic factors: Progesterone Receptor (PR), tumor stage, age at diagnosis (dx) and category of endocrine therapy (aromatase inhibitor, AI; tamoxifen, Tam). Boxes indicate the hazard ratio and error bars indicate the 95% confidence intervals. A yellow box indicates a statistically significant higher hazard ratio, and a blue box indicates a statistically significant lower hazard ratio relative to reference. **(C-D)** Kaplan-Meier survival curves measuring the specified outcomes in patients with mutations in specified cell cycle checkpoint kinase genes in breast (**C**) and across non-breast cancer types (**D**). Log rank test determined p-values. HR, hazard ratios; ns, not significant; $p \leq 0.1^{\#}$, $p \leq 0.05^{*}$, $p \leq 0.01^{**}$, $p \leq 0.001^{***}$. Supports data presented in **Figure 8**.

Supplementary Methods

Details of datasets

MSKCC, Cancer Cell 2019 (*17*): Clinical and mutational data (*ESR1, TP53, ATM, ATR, CHEK2* and *CHEK1*) collected from 1756 patients with primary hormone receptor positive (i.e. ER and/or PR+) (HR+)/HER2- (n=1365), HR-/HER2+ (n=58), TNBC (n=168) and HR+/HER2+ (n=165) breast cancers.

TCGA, Nature 2012 (*86*): Downloaded from cBioPortal (*87*) for clinical and mutational (*ESR1*,*TP53*, *ATM*, *ATR*, *CHEK2* and *CHEK1*) analysis in December 2019.The data set is composed of clinical and mutational data collected from 825 patients with primary HR+/HER2- (n=486), HR-/HER2+ (n=31), TNBC (n=123), HR+/HER2+ (n=79) and undetermined (n=106) breast cancers.

METABRIC Nature 2012 (*49*) *& Nature Communication 2016* (*88*)*:* Downloaded from cBioPortal for clinical and mutational (*ESR1*, *TP53*, *ATR* and *CHEK2*) analysis in December 2019.The data set is composed of clinical and mutational data collected from 2509 patients with primary HR+/HER2- (n=1398), HR-/HER2+ (n=139), TNBC (n=320), HR+/HER2+ (n=108) and undetermined (n=544) breast cancers.

Broad, Nature 2012 (*71*): Downloaded from cBioPortal for clinical and mutational (*ESR1, TP53, ATM, ATR, CHEK2* and *CHEK1*) analysis in March 2020.The data set is composed of clinical and mutational data collected from 103 patients with primary HR+/HER2- (n=37), HR-/HER2+ (n=6), TNBC (n=6), HR+/HER2+ (n=2) and undetermined (n=52) breast cancers.

MBCP, Provisional, February 2020 (*89*): Downloaded from cBioPortal for clinical and mutational (*ESR1, TP53, ATM, ATR, CHEK2* and *CHEK1*) analysis in March 2020.The data set is composed of clinical and mutational data collected from 180 metastatic patients with

HR+/HER2- (n=50), HR-/HER2+ (n=8), TNBC (n=8), HR+/HER2+ (n=21) and undetermined (n=93) breast cancers.

British Columbia, Nature 2012 (*90*)*:* Downloaded from cBioPortal for clinical and mutational (*ESR1, TP53, ATM, ATR, CHEK2* and *CHEK1*) analysis in March 2020.The data set is composed of clinical and mutational data collected from 107 patients with primary HR+/HER2- (n=8), TNBC (n=90) and undetermined (n=9) breast cancers.

<u>Pan-cancer analyses</u>

Details of Datasets:

MSKCC pan-cancer dataset, Nature Medicine 2017: Downloaded from cBioPortal for clinical and mutational analysis (*TP53*, *ATM*, *ATR*, *CHEK2*) in March 2023. The dataset is composed of clinical and mutational data collected from 10,336 primary and metastatic cancer patients across cancer types including at least 100 samples each from patients with non-small cell lung cancer (n=1668), breast cancer (n=1337), colorectal cancer (n=1007), prostate cancer (n=717), glioma (n=553), pancreatic cancer (n=502), soft tissue sarcoma (n=443), bladder (n=423), melanoma (n=365), renal cell carcinoma (n=361), hepatobiliary cancer (n=355), and esophagogastric cancer (n=341). All breast cancer samples were excluded from pan-cancer analyses.

TCGA pan-cancer dataset (cBioPortal): Downloaded for clinical mutation analysis (*TP53*, *ATM*, *ATR*, *CHEK1*, *CHEK2*) in March 2023. The dataset is composed of clinical and mutational data collected from 10, 953 primary samples from cancer patients with a range of cancer types including at least 100 samples each from patients with breast cancer (n=1,084), non-small cell lung cancer (n=1,053), esophagogastric cancer (n=622), colorectal cancer (n=594), glioblastoma (n=592), endometrial cancer (n=586), ovarian cancer (n=585), head and neck cancer (n=523), glioma (n=514), renal clear cell carcinoma (n=512), thyroid cancer (n=500), and prostate cancer

(n=494). All breast cancer samples were excluded from pan-cancer analyses. Main figure Kaplan-Meier survival curves analyze data solely from samples from women, while supplementary figures show Kaplan-Meier analyses of the entire dataset with no subsetting.

China pan-cancer dataset (cBioPortal): Downloaded for clinical mutation analysis (*TP53*, *ATM*, *ATR*, *CHEK1*, *CHEK2*) in March 2023. The dataset is composed of clinical and mutational data collected from 10, 9 primary samples from cancer patients with a range of cancer types including at least 100 samples each from patients with non-small cell lung cancer (n=2,039), colorectal cancer (n=1,225), hepatocellular carcinoma (n=1,133), gastric cancer (n=866), esophageal carcinoma (n=610), soft tissue sarcoma (n=571), intrahepatic cholangiocarcinoma (n=555), pancreatic cancer (n=498), extrahepatic cholangiocarcinoma (n=351), breast carcinoma (n=323), renal cell carcinoma (n=308), and ovarian cancer (n=261).

Survival analyses

Kaplan-Meier survival curves and log rank tests were used to test the association of mutations in cell cycle checkpoint kinase genes with disease-specific survival as provided by the TCGA data in cBioPortal. For analyses presented in main figures, only association of survival outcomes in women was considered while the associated supplementary figure includes all patients in the dataset. Hazard ratios were calculated using cox regression analyses including the parameters of cancer type, sex, age at diagnosis and AJCC-defined metastasis stage.

VCD-induced menopause Female mice were genotyped at 5 weeks, housed in random groups at 10 weeks, and started on estrogen treatment. VCD (Sigma-Aldrich Cat#S453005) injections began when mice were 12 weeks old and administered IP at a dosage of 160 mg/kg. Control mice were injected with PBS in sesame oil. Mice were harvested 8 weeks post injection, and serum collected via cardiac puncture. Ligand Core at UVA performed estradiol analysis.

REFERENCES AND NOTES

1. R. T. Abraham, Cell cycle checkpoint signaling through the ATM and ATR kinases. *Genes Dev.* **15**, 2177–2196 (2001).

2. J. Bartek, J. Lukas, Chk1 and Chk2 kinases in checkpoint control and cancer. *Cancer Cell* **3**, 421–429 (2003).

3. H. L. Smith, H. Southgate, D. A. Tweddle, N. J. Curtin, DNA damage checkpoint kinases in cancer. *Expert Rev. Mol. Med.* **22**, e2 (2020).

4. A. Sancar, L. A. Lindsey-Boltz, K. Unsal-Kaçmaz, S. Linn, Molecular mechanisms of mammalian DNA repair and the DNA damage checkpoints. *Annu. Rev. Biochem.* **73**, 39–85 (2004).

5. C.-M. Aliouat-Denis, N. Dendouga, I. Van den Wyngaert, H. Goehlmann, U. Steller, I. van de Weyer, N. Van Slycken, L. Andries, S. Kass, W. Luyten, M. Janicot, J. E. Vialard, p53-independent regulation of p21Waf1/Cip1 expression and senescence by Chk2. *Mol. Cancer Res.* **3**, 627–634 (2005).

6. S. M. de Toledo, E. I. Azzam, W. K. Dahlberg, T. B. Gooding, J. B. Little, ATM complexes with HDM2 and promotes its rapid phosphorylation in a p53-independent manner in normal and tumor human cells exposed to ionizing radiation. *Oncogene* **19**, 6185–6193 (2000).

7. G. G. Jones, P. M. Reaper, A. R. Pettitt, P. D. Sherrington, The ATR-p53 pathway is suppressed in noncycling normal and malignant lymphocytes. *Oncogene* **23**, 1911–1921 (2004).

8. K. D. Fagan-Solis, D. A. Simpson, R. J. Kumar, L. G. Martelotto, L. E. Mose, N. U. Rashid, A. Y. Ho, S. N. Powell, Y. H. Wen, J. S. Parker, J. S. Reis-Filho, J. H. J. Petrini, G. P. Gupta, A P53-independent DNA damage response suppresses oncogenic proliferation and genome instability. *Cell Rep.* **30**, 1385–1399.e7 (2020).

9. R. Buisson, J. L. Boisvert, C. H. Benes, L. Zou, Distinct but concerted roles of ATR, DNA-PK, and Chk1 in countering replication stress during S phase. *Mol. Cell* **59**, 1011–1024 (2015).

10. G. K. Dasika, S. C. J. Lin, S. Zhao, P. Sung, A. Tomkinson, E. Y. H. P. Lee, DNA damage-induced cell cycle checkpoints and DNA strand break repair in development and tumorigenesis. *Oncogene* **18**, 7883–

7899 (1999).

11. Global, Regional, and National Cancer Incidence,Mortality, years of life lost, years lived with disability, and disability-adjusted life-years for 29 cancer groups, 1990 to 2017: A systematic analysis for the global burden of disease study (JAMA, 2019); https://jamanetwork.com/journals/jamaoncology/fullarticle/2752381.

12. C. M. Perou, T. Sørlie, M. B. Eisen, M. van de Rijn, S. S. Jeffrey, C. A. Rees, J. R. Pollack, D. T. Ross, H. Johnsen, L. A. Akslen, Ø. Fluge, A. Pergamenschikov, C. Williams, S. X. Zhu, P. E. Lønning, A.L. Børresen-Dale, P. O. Brown, D. Botstein, Molecular portraits of human breast tumours. *Nature* **406**, 747–752 (2000).

13. D. J. Slamon, G. M. Clark, S. G. Wong, W. J. Levin, A. Ullrich, W. L. McGuire, Human breast cancer: Correlation of relapse and survival with amplification of the HER-2/neu oncogene. *Science* **235**, 177–182 (1987).

14. J. T. Lei, M. Anurag, S. Haricharan, X. Gou, M. J. Ellis, Endocrine therapy resistance: New insights. *Breast* **48** Suppl 1, S26–S30 (2019).

15. B. Huang, M. Warner, J.-Å. Gustafsson, Estrogen receptors in breast carcinogenesis and endocrine therapy. *Mol. Cell. Endocrinol.*, 418 (2015).

16. A. B. Hanker, D. R. Sudhan, C. L. Arteaga, Overcoming endocrine resistance in breast cancer. *Cancer Cell* **37**, 496–513 (2020).

17. P. Razavi, M. T. Chang, G. Xu, C. Bandlamudi, D. S. Ross, N. Vasan, Y. Cai, C. M. Bielski, M. T.A. Donoghue, P. Jonsson, A. Penson, R. Shen, F. Pareja, R. Kundra, S. Middha, M. L. Cheng, A. Zehir, C. Kandoth, R. Patel, K. Huberman, L. M. Smyth, K. Jhaveri, S. Modi, T. A. Traina, C. Dang, W. Zhang, B. Weigelt, B. T. Li, M. Ladanyi, D. M. Hyman, N. Schultz, M. E. Robson, C. Hudis, E. Brogi, A. Viale, L. Norton, M. N. Dickler, M. F. Berger, C. A. Iacobuzio-Donahue, S. Chandarlapaty, M. Scaltriti, J. S. Reis-Filho, D. B. Solit, B. S. Taylor, J. Baselga, The genomic landscape of endocrine-resistant advanced breast cancers. *Cancer Cell* **34**, 427–438.e6 (2018).

18. D. J. Slamon, B. Leyland-Jones, S. Shak, H. Fuchs, V. Paton, A. Bajamonde, T. Fleming, W. Eiermann,

J. Wolter, M. Pegram, J. Baselga, L. Norton, Use of chemotherapy plus a monoclonal antibody against HER2 for metastatic breast cancer that overexpresses HER2. *N. Engl. J. Med.* **344**, 783–792 (2001).

19. M. Anurag, S. Haricharan, M. J. Ellis, CDK4/6 inhibitor biomarker research: Are we barking up the wrong tree? *Clin. Cancer Res.* **26**, 3–5 (2020).

20. E. A. Musgrove, R. Hui, K. J. Sweeney, C. K. Watts, R. L. Sutherland, Cyclins and breast cancer. *J. Mammary Gland Biol. Neoplasia* **1**, 153–162 (1996).

21. M. C. Southey, D. E. Goldgar, R. Winqvist, K. Pylkäs, F. Couch, M. Tischkowitz, W. D. Foulkes, J. Dennis, K. Michailidou, E. J. van Rensburg, T. Heikkinen, H. Nevanlinna, J. L. Hopper, T. Dörk, K. B.M. Claes, J. Reis-Filho, Z. L. Teo, P. Radice, I. Catucci, P. Peterlongo, H. Tsimiklis, F. A. Odefrey, J. G. Dowty, M. K. Schmidt, A. Broeks, F. B. Hogervorst, S. Verhoef, J. Carpenter, C. Clarke, R. J. Scott, P. A. Fasching, L. Haeberle, A. B. Ekici, M. W. Beckmann, J. Peto, I. dos-Santos-Silva, O. Fletcher, N. Johnson, M. K. Bolla, E. J. Sawyer, I. Tomlinson, M. J. Kerin, N. Miller, F. Marme, B. Burwinkel, R. Yang, P. Guénel, T. Truong, F. Menegaux, M. Sanchez, S. Bojesen, S. F. Nielsen, H. Flyger, J. Benitez, M. P. Zamora, J. I. A. Perez, P. Menéndez, H. Anton-Culver, S. Neuhausen, A. Ziogas, C. A. Clarke, H. Brenner, V. Arndt, C. Stegmaier, H. Brauch, T. Brüning, Y. D. Ko, T. A. Muranen, K. Aittomäki, C. Blomqvist, N. V. Bogdanova, N. N. Antonenkova, A. Lindblom, S. Margolin, A. Mannermaa, V. Kataja, V. M. Kosma, J. M. Hartikainen, A. B. Spurdle; kConFab Investigators; Australian Ovarian Cancer Study Group, E. Wauters, D. Smeets, B. Beuselinck, G. Floris, J. Chang-Claude, A. Rudolph, P. Seibold, D. Flesch-Janys, J. E. Olson, C. Vachon, V. S. Pankratz, C. McLean, C. A. Haiman, B. E. Henderson, F. Schumacher, L. le Marchand, V. Kristensen, G. G. Alnæs, W. Zheng, D. J. Hunter, S. Lindstrom, S. E. Hankinson, P. Kraft, I. Andrulis, J. A. Knight, G. Glendon, A. M. Mulligan, A. Jukkola-Vuorinen, M. Grip, S. Kauppila, P. Devilee, R. A. E. M. Tollenaar, C. Seynaeve, A. Hollestelle, M. Garcia-Closas, J. Figueroa, S. J. Chanock, J. Lissowska, K. Czene, H. Darabi, M. Eriksson, D. M. Eccles, S. Rafiq, W. J. Tapper, S. M. Gerty, M. J. Hooning, J. W. M. Martens, J. M. Collée, M. Tilanus-Linthorst, P. Hall, J. Li, J. S. Brand, K. Humphreys, A. Cox, M. W. R. Reed, C. Luccarini, C. Baynes, A. M. Dunning, U. Hamann, D. Torres, H. U. Ulmer, T. Rüdiger, A. Jakubowska, J. Lubinski, K. Jaworska, K. Durda, S. Slager, A. E. Toland, C. B. Ambrosone, D. Yannoukakos, A. Swerdlow, A. Ashworth, N. Orr, M. Jones, A. González-Neira, G. Pita, M. R. Alonso, N. Álvarez, D. Herrero, D. C. Tessier, D. Vincent, F. Bacot, J. Simard, M. Dumont, P. Soucy, R. Eeles, K. Muir, F. Wiklund, H.

Gronberg, J. Schleutker, B. G. Nordestgaard, M. Weischer, R. C. Travis, D. Neal, J. L. Donovan, F. C. Hamdy, K.T. Khaw, J. L. Stanford, W. J. Blot, S. Thibodeau, D. J. Schaid, J. L. Kelley, C. Maier, A. S. Kibel, C. Cybulski, L. Cannon-Albright, K. Butterbach, J. Park, R. Kaneva, J. Batra, M. R. Teixeira, Z. Kote-Jarai, A. A. A. Olama, S. Benlloch, S. P. Renner, A. Hartmann, A. Hein, M. Ruebner, D. Lambrechts, E. van Nieuwenhuysen, I. Vergote, S. Lambretchs, J. A. Doherty, M. A. Rossing, S. Nickels, U. Eilber, S. Wang-Gohrke, K. Odunsi, L. E. Sucheston-Campbell, G. Friel, G. Lurie, J. L. Killeen, L. R. Wilkens, M. T. Goodman, I. Runnebaum, P. A. Hillemanns, L. M. Pelttari, R. Butzow, F. Modugno, R. P. Edwards, R. B. Ness, K. B. Moysich, A. du Bois, F. Heitz, P. Harter, S. Kommoss, B. Y. Karlan, C. Walsh, J. Lester, A. Jensen, S. K. Kjaer, E. Høgdall, B. Peissel, B. Bonanni, L. Bernard, E. L. Goode, B. L. Fridley, R. A. Vierkant, J. M. Cunningham, M. C. Larson, Z. C. Fogarty, K. R. Kalli, D. Liang, K. H. Lu, M. A. T. Hildebrandt, X. Wu, D. A. Levine, F. Dao, M. Bisogna, A. Berchuck, E. S. Iversen, J. R. Marks, L. Akushevich, D. W. Cramer, J. Schildkraut, K. L. Terry, E. M. Poole, M. Stampfer, S. S. Tworoger, E. V. Bandera, I. Orlow, S. H. Olson, L. Bjorge, H. B. Salvesen, A. M. van Altena, K. K. H. Aben, L. A. Kiemeney, L. F. A. G. Massuger, T. Pejovic, Y. Bean, A. Brooks-Wilson, L. E. Kelemen, L. S. Cook, N. D. le, B. Górski, J. Gronwald, J. Menkiszak, C. K. Høgdall, L. Lundvall, L. Nedergaard, S. A. Engelholm, E. Dicks, J. Tyrer, I. Campbell, I. McNeish, J. Paul, N. Siddiqui, R. Glasspool, A. S. Whittemore, J. H. Rothstein, V. McGuire, W. Sieh, H. Cai, X.O. Shu, R. T. Teten, R. Sutphen, J. R. McLaughlin, S. A. Narod, C. M. Phelan, A. N. Monteiro, D. Fenstermacher, H.Y. Lin, J. B. Permuth, T. A. Sellers, Y. A. Chen, Y.Y. Tsai, Z. Chen, A. Gentry-Maharaj, S. A. Gayther, S. J. Ramus, U. Menon, A. H. Wu, C. L. Pearce, D. van den Berg, M. C. Pike, A. Dansonka-Mieszkowska, J. Plisiecka-Halasa, J. Moes-Sosnowska, J. Kupryjanczyk, P. D.P. Pharoah, H. Song, I. Winship, G. Chenevix-Trench, G. G. Giles, S. V. Tavtigian, D. F. Easton, R. L. Milne, PALB2, CHEK2 and ATM rare variants and cancer risk: Data from COGS. *J. Med. Genet.* **53**, 800–811 (2016).

22. B. Decker, J. Allen, C. Luccarini, K. A. Pooley, M. Shah, M. K. Bolla, Q. Wang, S. Ahmed, C. Baynes, D. M. Conroy, J. Brown, R. Luben, E. A. Ostrander, P. D.P. Pharoah, A. M. Dunning, D. F. Easton, Rare, protein-truncating variants in ATM, CHEK2 and PALB2, but not XRCC2, are associated with increased breast cancer risks. *J. Med. Genet.* **54**, 732–741 (2017).

23. L. S. Stucci, V. Internò, M. Tucci, M. Perrone, F. Mannavola, R. Palmirotta, C. Porta, The ATM gene in breast cancer: Its relevance in clinical practice. *Genes* **12**, 727 (2021).

24. N. Tung, N. U. Lin, J. Kidd, B. A. Allen, N. Singh, R. J. Wenstrup, A. R. Hartman, E. P. Winer, J. E. Garber, Frequency of germline mutations in 25 cancer susceptibility genes in a sequential series of patients with breast cancer. *J. Clin. Oncol.* **34**, 1460–1468 (2016).

25. M. Choi, T. Kipps, R. Kurzrock, ATM mutations in cancer: Therapeutic implications. *Mol. Cancer Ther.* **15**, 1781–1791 (2016).

26. S. Haricharan, N. Punturi, P. Singh, K. R. Holloway, M. Anurag, J. Schmelz, C. Schmidt, J. T. Lei, V. Suman, K. Hunt, J. A. Olson Jr., J. Hoog, S. Li, S. Huang, D. P. Edwards, S. M. Kavuri, M. N. Bainbridge, C. X. Ma, M. J. Ellis, Loss of MutL disrupts Chk2-dependent cell-cycle control through CDK4/6 to promote intrinsic endocrine therapy resistance in primary breast cancer. *Cancer Discov.* **7**, 1168–1183 (2017).

27. M. Anurag, N. Punturi, J. Hoog, M.N. Bainbridge, M. J. Ellis, S. Haricharan, Comprehensive profiling of dna repair defects in breast cancer identifies a novel class of endocrine therapy resistance drivers. *Clin. Cancer Res.* **24**, 4887–4899 (2018).

28. A. Mazumder, A. Jimenez, R. E. Ellsworth, S. J. Freedland, S. George, M. N. Bainbridge, S. Haricharan, The DNA damage repair landscape in Black women with breast cancer. *Ther. Adv. Med. Oncol.* **14**, 17588359221075458 (2022).

29. T. M. A. Abdel-Fatah, A. Arora, N. Alsubhi, D. Agarwal, P. M. Moseley, C. Perry, R. Doherty, S. Y. T. Chan, A. R. Green, E. Rakha, G. Ball, I. O. Ellis, S. Madhusudan, Clinicopathological significance of ATM-chk2 expression in sporadic breast cancers: A comprehensive analysis in large cohorts. *Neoplasia* **16**, 982–991 (2014).

30. K. Krug, E. J. Jaehnig, S. Satpathy, L. Blumenberg, A. Karpova, M. Anurag, G. Miles, P. Mertins, Y. Geffen, L. C. Tang, D. I. Heiman, S. Cao, Y. E. Maruvka, J. T. Lei, C. Huang, R. B. Kothadia, A. Colaprico, C. Birger, J. Wang, Y. Dou, B. Wen, Z. Shi, Y. Liao, M. Wiznerowicz, M. A. Wyczalkowski, X. S. Chen, J. J. Kennedy, A. G. Paulovich, M. Thiagarajan, C. R. Kinsinger, T. Hiltke, E. S. Boja, M. Mesri, A. I. Robles, H. Rodriguez, T. F. Westbrook, L. Ding, G. Getz, K. R. Clauser, D. Fenyö, K. V. Ruggles, B. Zhang, D.R. Mani, S. A. Carr, M. J. Ellis, M. A. Gillette, S. C. Avanessian, S. Cai, D. Chan, X. Chen, N. J. Edwards, A. N. Hoofnagle, M. H. Kane, K. A. Ketchum, E. Kuhn, D. A. Levine, S. Li, D.

C. Liebler, T. Liu, J. Luo, S. Madhavan, C. Maher, J. E. McDermott, P. B. McGarvey, M. Oberti, A. Pandey, S. H. Payne, D. F. Ransohoff, R. C. Rivers, K. D. Rodland, P. Rudnick, M. E. Sanders, K. M. Shaw, I. M. Shih, R. J.C. Slebos, R. D. Smith, M. Snyder, S. E. Stein, D. L. Tabb, R. R. Thangudu, S. Thomas, Y. Wang, F. M. White, J. R. Whiteaker, G. A. Whiteley, H. Zhang, Z. Zhang, Y. Zhao, H. Zhu, L. J. Zimmerman, Proteogenomic landscape of breast cancer tumorigenesis and targeted therapy. *Cell* **183**, 1436–1456.e31 (2020).

31. F. R. Day, D. J. Thompson, H. Helgason, D. I. Chasman, H. Finucane, P. Sulem, K. S. Ruth, S. Whalen, A. K. Sarkar, E. Albrecht, E. Altmaier, M. Amini, C. M. Barbieri, T. Boutin, A. Campbell, E. Demerath, A. Giri, C. He, J. J. Hottenga, R. Karlsson, I. Kolcic, P.-R. Loh, K. L. Lunetta, M. Mangino, B. Marco, G. M. Mahon, S. E. Medland, I. M. Nolte, R. Noordam, T. Nutile, L. Paternoster, N. Perjakova, E. Porcu, L. M. Rose, K. E. Schraut, A. V. Segrè, A. V. Smith, L. Stolk, A. Teumer, I. L. Andrulis, S. Bandinelli, M. W. Beckmann, J. Benitez, S. Bergmann, M. Bochud, E. Boerwinkle, S. E. Bojesen, M. K. Bolla, J. S. Brand, H. Brauch, H. Brenner, L. Broer, T. Brüning, J. E. Buring, H. Campbell, E. Catamo, S. Chanock, G. Chenevix-Trench, T. Corre, F. J. Couch, D. L. Cousminer, A. Cox, L. Crisponi, K. Czene, G. D. Smith, Eco J C N de Geus, R. de Mutsert, I. De Vivo, J. Dennis, P. Devilee, Isabel Dos-Santos-Silva, A. M. Dunning, J. G. Eriksson, P. A. Fasching, L. Fernández-Rhodes, L. Ferrucci, D. Flesch-Janys, L. Franke, M. Gabrielson, I. Gandin, G. G. Giles, H. Grallert, D. F. Gudbjartsson, P. Guénel, P. Hall, E. Hallberg, U. Hamann, T. B. Harris, C. A. Hartman, G. Heiss, M. J. Hooning, J. L. Hopper, F. Hu, D. J. Hunter, M Arfan Ikram, H. K. Im, M.-R. Järvelin, P. K. Joshi, D. Karasik, M. Kellis, Z. Kutalik, G. L. Chance, D. Lambrechts, C. Langenberg, L. J. Launer, J. S. E. Laven, S. Lenarduzzi, J. Li, P. A. Lind, S. Lindstrom, Y. M. Liu, Jian'an Luan, R. Mägi, A. Mannermaa, H. Mbarek, M. I. McCarthy, C. Meisinger, T. Meitinger, C. Menni, A. Metspalu, K. Michailidou, L. Milani, R. L. Milne, G. W. Montgomery, A. M. Mulligan, M. A. Nalls, P. Navarro, H. Nevanlinna, D. R. Nyholt, A. J. Oldehinkel, T. A .O'Mara, S. Padmanabhan, A. Palotie, N. Pedersen, A. Peters, J. Peto, P. D. P. Pharoah, A. Pouta, P. Radice, I. Rahman, S. M. Ring, A. Robino, F. R. Rosendaal, I. Rudan, R. Rueedi, D. Ruggiero, C. F. Sala, M. K. Schmidt, R. A. Scott, M. Shah, R. Sorice, M. C. Southey, U. Sovio, M. Stampfer, M. Steri, K. Strauch, T. Tanaka, E. Tikkanen, N. J. Timpson, M. Traglia, T. Truong, J. P. Tyrer, A. G. Uitterlinden, D. R. Velez Edwards, V. Vitart, U. Völker, P. Vollenweider, Q. Wang, E. Widen, K. W. van Dijk, G. Willemsen, R. Winqvist, B. H. R. Wolffenbuttel, J. H. Zhao, M. Zoledziewska, M. Zygmunt, B. Z. Alizadeh, D. I. Boomsma, M. Ciullo, F. Cucca, T. Esko, N.

Franceschini, C. Gieger, V. Gudnason, C. Hayward, P. Kraft, D. A. Lawlor, P. K. E. Magnusson, N. G. Martin, D. O. Mook-Kanamori, E. A. Nohr, O. Polasek, D. Porteous, A. L. Price, P. M. Ridker, H. Snieder, T. D. Spector, D. Stöckl, D. Toniolo, S. Ulivi, J. A. Visser, H. Völzke, N. J. Wareham, J. F. Wilson; Life Lines Cohort Study, InterAct Consortium, kConFab/AOCS Investigators; Endometrial Cancer Association Consortium; Ovarian Cancer Association Consortium; PRACTICAL consortium, A. B. Spurdle, U. Thorsteindottir, K. S. Pollard, D. F. Easton, J. Y. Tung, J. Chang-Claude, D. Hinds, A. Murray, J. M. Murabito, K. Stefansson, K. K. Ong, J. R. B. Perry, Genomic analyses identify hundreds of variants associated with age at menarche and support a role for puberty timing in cancer risk. *Nat. Genet.* **49**, 834–841 (2017).

32. K. J. Lee, E. Mann, G. Wright, C. G. Piett, Z. D. Nagel, N. R. Gassman, Exploiting DNA repair defects in triple negative breast cancer to improve cell killing. *Ther. Adv. Med. Oncol.* **12**, 1758835920958354 (2020).

33. C. X. Ma, S. Cai, S. Li, C. E. Ryan, Z. Guo, W. T. Schaiff, L. Lin, J. Hoog, R. J. Goiffon, A. Prat, R. L. Aft, M. J. Ellis, H. Piwnica-Worms, Targeting Chk1 in p53-deficient triple-negative breast cancer is therapeutically beneficial in human-in-mouse tumor models. *J. Clin. Invest.* **122**, 1541–1552 (2012).

34. Z. Wilson, R. Odedra, Y. Wallez, P. W. G. Wijnhoven, A. M. Hughes, J. Gerrard, G. N. Jones, H. Bargh-Dawson, E. Brown, L. A. Young, M. J. O'Connor, A. Lau, ATR inhibitor AZD6738 (ceralasertib) exerts antitumor activity as a monotherapy and in combination with chemotherapy and the PARP inhibitor olaparib. *Cancer Res.* **82**, 1140–1152 (2022)..

35. S. da Costa E Silva Carvalho, N. M. Cury, D. B. Brotto, L. F. de Araujo, R. C. A. Rosa, L. A. Texeira, J. R. Plaça, A. A. Marques, K. C. Peronni, P. de Cássia Ruy, G. A. Molfetta, J. C. Moriguti, D. M. Carraro, E. I. Palmero, P. Ashton-Prolla, V. E. de Faria Ferraz, W. A. Silva Jr., Germline variants in DNA repair genes associated with hereditary breast and ovarian cancer syndrome: Analysis of a 21 gene panel in the Brazilian *population. Genomics* **13**, 21 (2020).

36. F. Durocher, Y. Labrie, P. Soucy, O. Sinilnikova, D. Labuda, P. Bessette, J. Chiquette, R. Laframboise, J. Lépine, B. Lespérance, G. Ouellette, R. Pichette, M. Plante, S. V. Tavtigian, J. Simard, Mutation analysis and characterization of ATR sequence variants in breast cancer cases from high-risk French Canadian breast/ovarian cancer families. *BMC Cancer* **6**, 230 (2006).

37. D. Mandelker, R. Kumar, X. Pei, P. Selenica, J. Setton, S. Arunachalam, O. Ceyhan-Birsoy, D. N. Brown, L. Norton, M. E. Robson, H. Y. Wen, S. Powell, N. Riaz, B. Weigelt, J. S. Reis-Filho, The landscape of somatic genetic alterations in breast cancers from CHEK2 germline mutation carriers. *JNCI Cancer Spectr.* **3**, pkz027 (2019).

38. C. Cortes-Urrea, F. Bueno-Gutiérrez, M. Solarte, M. Guevara-Burbano, F. Tobar-Tosse, P. E. Vélez-Varela, J. C. Bonilla, G. Barreto, J. Velasco-Medina, P. A. Moreno, J. de Las Rivas, Exomes of ductal luminal breast cancer patients from Southwest Colombia: Gene mutational profile and related expression alterations. *Biomolecules* **10**, E698 (2020).

39. Z. K. Stadler, A. Maio, D. Chakravarty, Y. Kemel, M. Sheehan, E. Salo-Mullen, K. Tkachuk, C. J. Fong, B. Nguyen, A. Erakky, K. Cadoo, Y. Liu, M. I. Carlo, A. Latham, H. Zhang, R. Kundra, S. Smith, J. Galle, C. Aghajanian, N. Abu-Rustum, A. Varghese, E. M. O'Reilly, M. Morris, W. Abida, M. Walsh, A. Drilon, G. Jayakumaran, A. Zehir, M. Ladanyi, O. Ceyhan-Birsoy, D. B. Solit, N. Schultz, M. F. Berger, D. Mandelker, L. A. Diaz Jr., K. Offit, M. E. Robson, Therapeutic implications of germline testing in patients with advanced cancers. *JCO* **39**, 2698–2709 (2021).

40. E. M. Bahassi, C. G. Penner, S. B. Robbins, E. Tichy, E. Feliciano, M. Yin, L. Liang, L. Deng, J. A. Tischfield, P. J. Stambrook, The breast cancer susceptibility allele CHEK2*1100delC promotes genomic instability in a knock-in mouse model. *Mutat. Res.* **616**, 201–209 (2007).

41. H. L. Brooks, D. P. Pollow, P. B. Hoyer, The VCD mouse model of menopause and perimenopause for the study of sex differences in cardiovascular disease and the metabolic syndrome. *Physiology (Bethesda)* **31**, 250–257 (2016).

42. S. E. Meyer, B. E. Peace, E. M. Bahassi, G. M. Kavanaugh, P. K. Wagh, S. B. Robbins, M. Yin, S. I. Wells, G. M. Zinser, P. J. Stambrook, S. E. Waltz, Chk2*1100delC acts in synergy with the ron receptor tyrosine kinase to accelerate mammary tumorigenesis in mice. *Cancer Lett.* **296**, 186–193 (2010).

43. R. Jeselsohn, G. Buchwalter, C. De Angelis, M. Brown, R. Schiff, ESR1 mutations—A mechanism for acquired endocrine resistance in breast cancer. *Nat. Rev. Clin. Oncol.* **12**, 573–583 (2015).

44. D. R. Robinson, Y. M. Wu, P. Vats, F. Su, R. J. Lonigro, X. Cao, S. Kalyana-Sundaram, R. Wang, Y.

Ning, L. Hodges, A. Gursky, J. Siddiqui, S. A. Tomlins, S. Roychowdhury, K. J. Pienta, S. Y. Kim, J. S. Roberts, J. M. Rae, C. H. van Poznak, D. F. Hayes, R. Chugh, L. P. Kunju, M. Talpaz, A. F. Schott, A. M. Chinnaiyan, Activating ESR1 mutations in hormone-resistant metastatic breast cancer. *Nat. Genet.* **45**, 1446–1451 (2013).

45. S. Fan, Q. Meng, J. Xu, Y. Jiao, L. Zhao, X. Zhang, F. H. Sarkar, M. L. Brown, A. Dritschilo, E. M. Rosen, DIM (3,3′-diindolylmethane) confers protection against ionizing radiation by a unique mechanism. *PNAS* **110**, 18650–18655 (2013).

46. P. K. Kandala, S. K. Srivastava, Activation of checkpoint kinase 2 by 3,3′-diindolylmethane is required for causing G2/M cell cycle arrest in human ovarian cancer cells. *Mol. Pharmacol.* **78**, 297–309 (2010).

47. V. E. Anderson, M. I. Walton, P. D. Eve, K. J. Boxall, L. Antoni, J. J. Caldwell, W. Aherne, L. H. Pearl, A. W. Oliver, I. Collins, M. D. Garrett, CCT241533 is a potent and selective inhibitor of CHK2 that potentiates the cytotoxicity of PARP inhibitors. *Cancer Res.* **71**, 463–472 (2011).

48. J. Buendia-Buendia, O. Cohen, D. Kim, E. Jain, E. P. Winer, N. U. Lin, N. Wagle, Characterization of mutational processes in ER^+ metastatic breast cancer. *J. Clin. Oncol.* **37**, 1019–1019 (2019).

49. C. Curtis, S. P. Shah, S.-F. Chin, G. Turashvili, O. M. Rueda, M. J. Dunning, D. Speed, A. G. Lynch, S. Samarajiwa, Y. Yuan, S. Gräf, G. Ha, G. Haffari, A. Bashashati, R. Russell, S. M. Kinney; METABRIC Group, A. Langerød, A. Green, E. Provenzano, G. Wishart, S. Pinder, P. Watson, F. Markowetz, L. Murphy, I. Ellis, A. Purushotham, A.-L. Børresen-Dale, J. D. Brenton, S. Tavaré, C. Caldas, S. Aparicio, The genomic and transcriptomic architecture of 2,000 breast tumours reveals novel subgroups. *Nature* **486**, 346–352 (2012).

50. L. van Bijsterveldt, S. C. Durley, T. S. Maughan, T. C. Humphrey, The challenge of combining chemo- and radiotherapy with checkpoint kinase inhibitors. *Clin. Cancer Res.* **27**, 937–962 (2021).

51. M. K. Schmidt, R. A. E. M. Tollenaar, S. R. de Kemp, A. Broeks, C. J. Cornelisse, V. T. H. B. M. Smit, J. L. Peterse, F. E. van Leeuwen, L. J. van't Veer, Breast cancer survival and tumor characteristics in premenopausal women carrying the CHEK2*1100delC germline mutation. *J. Clin. Oncol.* **25**, 64–69 (2007).

52. K. Einarsdóttir, K. Humphreys, C. Bonnard, J. Palmgren, M. M. Iles, A. Sjölander, Y. Li, K. S. Chia, E. T. Liu, P. Hall, J. Liu, S. Wedrén, Linkage disequilibrium mapping of CHEK2: Common variation and breast cancer risk. *PLOS Med.* **3**, e168 (2006).

53. K. P. Lowry, H. A. Geuzinge, N. K. Stout, O. Alagoz, J. Hampton, K. Kerlikowske, H. J. de Koning, D. L. Miglioretti, N. T. van Ravesteyn, C. Schechter, B. L. Sprague, A. N. A. Tosteson, A. Trentham-Dietz, D. Weaver, M. J. Yaffe, J. M. Yeh, F. J. Couch, C. Hu, P. Kraft, E. C. Polley, J. S. Mandelblatt, A. W. Kurian, M. E. Robson; Breast Working Group of the Cancer Intervention and Surveillance Modeling Network (CISNET), in collaboration with the Breast Cancer Surveillance Consortium (BCSC), and the Cancer Risk Estimates Related to Susceptibility (CARRIERS) Consortium, S. N. Hart, K. L. Nathanson, S. M. Domchek, C. B. Ambrosone, H. Anton-Culver, P. Auer, E. V. Bandera, L. Berstein, K. A. Bertrand, E. S. Burnside, B. D. Carter, H. Eliassen, M. Gaudet, C. Haiman, J. M. Hodge, D. J. Hunter, E. J. Jacobs, E. M. John, C. Kooperberg, J. V. Lacey, L. le Marchand, S. Lindstrom, H. Ma, E. Martinez, S. Neuhausen, P. A. Newcomb, K. M. O'Brien, J. E. Olson, I. M. Ong, T. Pal, J. R. Palmer, A. V. Patel, S. Reid, L. Rosenberg, D. P. Sandler, R. Tamimi, J. A. Taylor, L. Teras, C. M. Vachon, C. Weinberg, S. Yadav, S. Yao, A. Ziogas, J. N. Weitzel, D. E. Goldgar, Breast cancer screening strategies for women with ATM, CHEK2, and PALB2 pathogenic variants: A comparative modeling analysis. *JAMA Oncol.* **8**, 587–596 (2022).

54. C. T. van Geelen, P. Savas, Z. L. Teo, S. J. Luen, C.F. Weng, Y. A. Ko, K. S. Kuykhoven, F. Caramia, R. Salgado, P. A. Francis, S. J. Dawson, S. B. Fox, A. Fellowes, S. Loi, Clinical implications of prospective genomic profiling of metastatic breast cancer patients. *Breast Cancer Res.* **22**, 91 (2020).

55. T. A. Muranen, C. Blomqvist, T. Dörk, A. Jakubowska, P. Heikkilä, R. Fagerholm, D. Greco, K. Aittomäki, S. E. Bojesen, M. Shah, A. M. Dunning, V. Rhenius, P. Hall, K. Czene, J. S. Brand, H. Darabi, J. Chang-Claude, A. Rudolph, B. G. Nordestgaard, F. J. Couch, S. N. Hart, J. Figueroa, M. García-Closas, P. A. Fasching, M. W. Beckmann, J. Li, J. Liu, I. L. Andrulis, R. Winqvist, K. Pylkäs, A. Mannermaa, V. Kataja, A. Lindblom, S. Margolin, J. Lubinski, N. Dubrowinskaja, M. K. Bolla, J. Dennis, K. Michailidou, Q. Wang, D. F. Easton, P. D. P. Pharoah, M. K. Schmidt, H. Nevanlinna, Patient survival and tumor characteristics associated with CHEK2:p.I157T - findings from the Breast Cancer Association Consortium. *Breast Cancer Res.* **18**, 98 (2016).

56. A. Katoch, D. Nayak, M. M. Faheem, A. Kumar, P. K. Sahu, A. P. Gupta, L. D. Kumar, A. Goswami, Natural podophyllotoxin analog 4DPG attenuates EMT and colorectal cancer progression via activation of checkpoint kinase 2. *Cell Death Discov.* **7**, 25 (2021).

57. D. Nayak, A. Kumar, S. Chakraborty, R. Rasool, H. Amin, A. Katoch, V. Gopinath, V. Mahajan, M. K. Zilla, B. Rah, S. G. Gandhi, A. Ali, L. D. Kumar, A. Goswami, Inhibition of Twist1-mediated invasion by Chk2 promotes premature senescence in p53-defective cancer cells. *Cell Death Differ.* **24**, 1275–1287 (2017).

58. I. Collins, M. D. Garrett, Targeting the cell division cycle in cancer: CDK and cell cycle checkpoint kinase inhibitors. *Curr. Opin. Pharmacol.* **5**, 366–373 (2005).

59. R. S. Finn, M. Martin, H. S. Rugo, S. Jones, S. A. Im, K. Gelmon, N. Harbeck, O. N. Lipatov, J. M. Walshe, S. Moulder, E. Gauthier, D. R. Lu, S. Randolph, V. Diéras, D. J. Slamon, Palbociclib and Letrozole in Advanced Breast Cancer. *N. Engl. J. Med.* **375**, 1925–1936 (2016).

60. S. Angèle, J. Hall, The ATM gene and breast cancer: Is it really a risk factor? *Mutat. Res.* **462**, 167–178 (2000).

61. W.-C. Chou, L.-Y. Hu, C.-N. Hsiung, C.-Y. Shen, Initiation of the ATM-Chk2 DNA damage response through the base excision repair pathway. *Carcinogenesis* **36**, 832–840 (2015).

62. J. Smith, L. M. Tho, N. Xu, D. A. Gillespie, The ATM-Chk2 and ATR-Chk1 pathways in DNA damage signaling and cancer. *Adv. Cancer Res.* **108**, 73–112 (2010).

63. X. Yue, C. Bai, D. Xie, T. Ma, P.-K. Zhou, DNA-PKcs: A Multi-Faceted Player in DNA Damage Response. *Front. Genet.* **11**, (2020).

64. I. García-Santisteban, A. Llopis, L. Krenning, J. Vallejo-Rodríguez, B. van den Broek, A. M. Zubiaga, R. H. Medema, Sustained CHK2 activity, but not ATM activity, is critical to maintain a G1 arrest after DNA damage in untransformed cells. *BMC Biol.* **19**, 35 (2021).

65. D. R. Smith, H. S. Goh, Overexpression of the c-myc proto-oncogene in colorectal carcinoma is associated with a reduced mortality that is abrogated by point mutation of the p53 tumor suppressor

gene. *Clin. Cancer Res.* **2**, 1049–1053 (1996).

66. S. Haricharan, P. Brown, TLR4 has a TP53-dependent dual role in regulating breast cancer cell growth. *Proc. Natl. Acad. Sci. U.S.A.* **112**, E3216–3225 (2015).

67. E. S. Knudsen, V. Kumarasamy, R. Nambiar, J. D. Pearson, P. Vail, H. Rosenheck, J. Wang, K. Eng, R. Bremner, D. Schramek, S. M. Rubin, A. L. Welm, A. K. Witkiewicz, CDK/cyclin dependencies define extreme cancer cell-cycle heterogeneity and collateral vulnerabilities. *Cell Rep.* **38**, 110448 (2022).

68. K.-L. Huang, R. J. Mashl, Y. Wu, D. I. Ritter, J. Wang, C. Oh, M. Paczkowska, S. Reynolds, M. A. Wyczalkowski, N. Oak, A. D. Scott, M. Krassowski, A. D. Cherniack, K. E. Houlahan, R. Jayasinghe, L. B. Wang, D. C. Zhou, D. Liu, S. Cao, Y. W. Kim, A. Koire, J. F. McMichael, V. Hucthagowder, T. B. Kim, A. Hahn, C. Wang, M. D. McLellan, F. al-Mulla, K. J. Johnson, O. Lichtarge, P. C. Boutros, B. Raphael, A. J. Lazar, W. Zhang, M. C. Wendl, R. Govindan, S. Jain, D. Wheeler, S. Kulkarni, J. F. Dipersio, J. Reimand, F. Meric-Bernstam, K. Chen, I. Shmulevich, S. E. Plon, F. Chen, L. Ding, Pathogenic germline variants in 10,389 adult cancers. *Cell* **173**, 355–370.e14 (2018).

69. M. R. Middleton, E. Dean, T. R. J. Evans, G. I. Shapiro, J. Pollard, B. S. Hendriks, M. Falk, I. Diaz-Padilla, R. Plummer, Phase 1 study of the ATR inhibitor berzosertib (formerly M6620, VX-970) combined with gemcitabine ± cisplatin in patients with advanced solid tumours. *Br. J. Cancer* **125**, 510–519 (2021).

70. S. N. Waqar, C. Robinson, A. J. Olszanski, A. Spira, M. Hackmaster, L. Lucas, L. Sponton, H. Jin, U. Hering, D. Cronier, M. Grinberg, A. Seithel-Keuth, I. Diaz-Padilla, J. Berlin, Phase I trial of ATM inhibitor M3541 in combination with palliative radiotherapy in patients with solid tumors. *Invest. New Drugs* **40**, 596–605 (2022).

71. S. Banerji, K. Cibulskis, C. Rangel-Escareno, K. K. Brown, S. L. Carter, A. M. Frederick, M. S. Lawrence, A. Y. Sivachenko, C. Sougnez, L. Zou, M. L. Cortes, J. C. Fernandez-Lopez, S. Peng, K. G. Ardlie, D. Auclair, V. Bautista-Piña, F. Duke, J. Francis, J. Jung, A. Maffuz-Aziz, R. C. Onofrio, M. Parkin, N. H. Pho, V. Quintanar-Jurado, A. H. Ramos, R. Rebollar-Vega, S. Rodriguez-Cuevas, S. L. Romero-Cordoba, S. E. Schumacher, N. Stransky, K. M. Thompson, L. Uribe-Figueroa, J. Baselga, R. Beroukhim, K. Polyak, D. C. Sgroi, A. L. Richardson, G. Jimenez-Sanchez, E. S. Lander, S. B. Gabriel,

L. A. Garraway, T. R. Golub, J. Melendez-Zajgla, A. Toker, G. Getz, A. Hidalgo-Miranda, M. Meyerson, Sequence analysis of mutations and translocations across breast cancer subtypes. *Nature* **486**, 405–409 (2012).

72. L. Wu, H. Yao, H. Chen, A. Wang, K. Guo, W. Gou, Y. Yu, X. Li, M. Yao, S. Yuan, F. Pang, J. Hu, L. Chen, W. Liu, J. Yao, S. Zhang, X. Dong, W. Wang, J. Hu, Q. Ling, S. Ding, Y. Wei, Q. Li, W. Cao, S. Wang, Y. di, F. Feng, G. Zhao, J. Zhang, L. Huang, J. Xu, W. Yan, Z. Tong, D. Jiang, T. Ji, Q. Li, L. Xu, H. He, L. Shang, J. Liu, K. Wang, D. Wu, J. Shen, Y. Liu, T. Zhang, C. Liang, Y. Wang, Y. Shang, J. Guo, G. Liang, S. Xu, J. Liu, K. Wang, M. Wang, Landscape of somatic alterations in large-scale solid tumors from an Asian population. *Nat. Commun.* **13**, 4264 (2022).

73. M. J. Romero-Aleshire, M. K. Diamond-Stanic, A. H. Hasty, P. B. Hoyer, H. L. Brooks, Loss of ovarian function in the VCD mouse-model of menopause leads to insulin resistance and a rapid progression into the metabolic syndrome. *Am. J. Physiol. Regul. Integr. Comp. Physiol.* **297**, R587–R592 (2009).

74. N. B. Punturi, S. Seker, V. Devarakonda, A. Mazumder, R. Kalra, C. H. Chen, S. Li, T. Primeau, M. J. Ellis, S. M. Kavuri, S. Haricharan, Mismatch repair deficiency predicts response to HER2 blockade in HER2-negative breast cancer. *Nat. Commun.* **12**, 2940 (2021).

75. S. Raghavan, P. Mehta, Y. Xie, Y. L. Lei, G. Mehta, Ovarian cancer stem cells and macrophages reciprocally interact through the WNT pathway to promote pro-tumoral and malignant phenotypes in 3D engineered microenvironments. *J. Immunother. Cancer* **7**, 190 (2019).

76. P. Mehta, C. Novak, S. Raghavan, M. Ward, G. Mehta, Self-renewal and CSCs in vitro enrichment: Growth as floating spheres. *Methods Mol. Biol.* **1692**, 61–75 (2018).

77. S. N. VandenHeuvel, H. A. Farris, D. A. Noltensmeyer, S. Roy, D. A. Donehoo, S. Kopetz, S. Haricharan, A. J. Walsh, S. Raghavan, Decellularized organ biomatrices facilitate quantifiable in vitro 3D cancer metastasis models. *Soft Matter* **18**, 5791–5806 (2022).

78. Breast Cancer Association Consortium, N. Mavaddat, L. Dorling, S. Carvalho, J. Allen, A. González-Neira, R. Keeman, M. K. Bolla, J. Dennis, Q. Wang, T. U. Ahearn, I. L. Andrulis, M. W. Beckmann, S.

Behrens, J. Benitez, M. Bermisheva, C. Blomqvist, N. V. Bogdanova, S. E. Bojesen, I. Briceno, T. Brüning, N. J. Camp, A. Campbell, J. E. Castelao, J. Chang-Claude, S. J. Chanock, G. Chenevix-Trench, H. Christiansen, K. Czene, T. Dörk, M. Eriksson, D Gareth Evans, P. A. Fasching, J. D. Figueroa, H. Flyger, M. Gabrielson, M. Gago-Dominguez, J. Geisler, G. G. Giles, P. Guénel, A. Hadjisavvas, E. Hahnen, P. Hall, U. Hamann, J. M. Hartikainen, M. Hartman, R. Hoppe, A. Howell, A. Jakubowska, A. Jung, E. K. Khusnutdinova, V. N. Kristensen, J. Li, S. H. Lim, A. Lindblom, M. A. Loizidou, A. Lophatananon, J. Lubinski, M. J. Madsen, A. Mannermaa, M. Manoochehri, S. Margolin, D. Mavroudis, R. L. Milne, N. A. M. Taib, A. Morra, K. Muir, N. Obi, A. Osorio, T.-W. Park-Simon, P. Peterlongo, P. Radice, E. Saloustros, E. J. Sawyer, R. K. Schmutzler, M. Shah, X. Sim, M. C. Southey, H. Thorne, I. Tomlinson, D. Torres, T. Truong, C. H. Yip, A. B. Spurdle, M. P. G. Vreeswijk, A. M. Dunning, M. García-Closas, P. D. P. Pharoah, A. Kvist, T. A. Muranen, H. Nevanlinna, S. H. Teo, P. Devilee, M. K. Schmidt, D. F. Easton, Pathology of tumors associated with pathogenic germline variants in 9 breast cancer susceptibility genes. *JAMA Oncol.* **8**, e216744 (2022).

79. P. J. Ho, A. J. Khng, H. W. Loh, W.K. Ho, C. H. Yip, N. A. Mohd-Taib, V. K. M. Tan, B. K. T. Tan, S. M. Tan, E. Y. Tan, S. H. Lim, S. Jamaris, Y. Sim, F. Y. Wong, J. Ngeow, E. H. Lim, M. C. Tai, E. A. Wijaya, S. C. Lee, C. W. Chan, S. A. Buhari, P. M. Y. Chan, J. J. C. Chen, J. C. M. Seah, W. P. Lee, C. W. Mok, G. H. Lim, E. Woo, S.W. Kim, J. W. Lee, M. H. Lee, S. K. Park, A. M. Dunning, D. F. Easton, M. K. Schmidt, S.H. Teo, J. Li, M. Hartman, Germline breast cancer susceptibility genes, tumor characteristics, and survival. *Genome Med.* **13**, 185 (2021).

80. C. Hu, E. C. Polley, S. Yadav, J. Lilyquist, H. Shimelis, J. Na, S. N. Hart, D. E. Goldgar, S. Shah, T. Pesaran, J. S. Dolinsky, H. LaDuca, F. J. Couch, The contribution of germline predisposition gene mutations to clinical subtypes of invasive breast cancer from a clinical genetic testing cohort. *J. Natl. Cancer Inst.* **112**, 1231–1241 (2020).

81. P. Domagala, D. Wokolorczyk, C. Cybulski, T. Huzarski, J. Lubinski, W. Domagala, Different CHEK2 germline mutations are associated with distinct immunophenotypic molecular subtypes of breast cancer. *Breast Cancer Res. Treat.* **132**, 937–945 (2012).

82. J. Dennis, J. P. Tyrer, L. C. Walker, K. Michailidou, L. Dorling, M. K. Bolla, Q. Wang, T. U. Ahearn, I. L. Andrulis, H. Anton-Culver, N. N. Antonenkova, V. Arndt, K. J. Aronson, L. E. B. Freeman, M. W.

Beckmann, S. Behrens, J. Benitez, M. Bermisheva, N. V. Bogdanova, S. E. Bojesen, H. Brenner, J. E. Castelao, J. Chang-Claude, G. Chenevix-Trench, C. L. Clarke; NBCS Collaborators, V. N. Kristensen, K. K. Sahlberg, A. L. Børresen-Dale, I. T. Gram, O. Engebråten, B. Naume, J. Geisler, G. I. G. Alnæs, J. M. Collée, CTS Consortium, J. Lacey, E. Martinez, F. J. Couch, A. Cox, S. S. Cross, K. Czene, P. Devilee, T. Dörk, L. Dossus, A. H. Eliassen, M. Eriksson, D. G. Evans, P. A. Fasching, J. Figueroa, O. Fletcher, H. Flyger, L. Fritschi, M. Gabrielson, M. Gago-Dominguez, M. García-Closas, G. G. Giles, A. González-Neira, P. Guénel, E. Hahnen, C. A. Haiman, P. Hall, A. Hollestelle, R. Hoppe, J.L. Hopper, A. Howell; ABCTB Investigators, C. Clarke, J. Carpenter, D. Marsh, R. Scott, R. Baxter, D. Yip, A. Davis, N. Pathmanathan, P. Simpson, D. Graham, M. Sachchithananthan; kConFab/AOCS Investigators, I. Campbell, A. de Fazio, S. Fox, J. Kirk, G. Lindeman, R. Milne, M. Southey, A. Spurdle, H. Thorne, A. Jager, A. Jakubowska, E.M. John, N. Johnson, M.E. Jones, A. Jung, R. Kaaks, R. Keeman, E. Khusnutdinova, C. M. Kitahara, Y. D. Ko, V. M. Kosma, S. Koutros, P. Kraft, V. N. Kristensen, K. Kubelka-Sabit, A. W. Kurian, J. V. Lacey, D. Lambrechts, N. L. Larson, M. Linet, A. Ogrodniczak, A. Mannermaa, S. Manoukian, S. Margolin, D. Mavroudis, R. L. Milne, T. A. Muranen, R. A. Murphy, H. Nevanlinna, J. E. Olson, H. Olsson, T.W. Park-Simon, C. M. Perou, P. Peterlongo, D. Plaseska-Karanfilska, K. Pylkäs, G. Rennert, E. Saloustros, D. P. Sandler, E. J. Sawyer, M. K. Schmidt, R. K. Schmutzler, R. Shibli, A. Smeets, P. Soucy, M. C. Southey, A. J. Swerdlow, R. M. Tamimi, J. A. Taylor, L. R. Teras, M. B. Terry, I. Tomlinson, M. A. Troester, T. Truong, C. M. Vachon, C. Wendt, R. Winqvist, A. Wolk, X. R. Yang, W. Zheng, A. Ziogas, J. Simard, A. M. Dunning, P. D. P. Pharoah, D. F. Easton, Rare germline copy number variants (CNVs) and breast cancer risk. *Commun. Biol.* **5**, 65 (2022).

83. A. Toss, E. Tenedini, C. Piombino, M. Venturelli, I. Marchi, E. Gasparini, E. Barbieri, E. Razzaboni, F. Domati, F. Caggia, G. Grandi, F. Combi, G. Tazzioli, M. Dominici, E. Tagliafico, L. Cortesi, Clinicopathologic profile of breast cancer in germline ATM and CHEK2 mutation carriers. *Genes (Basel)* **12**, 616 (2021).

84. A.-L. Renault, N. Mebirouk, L. Fuhrmann, G. Bataillon, E. Cavaciuti, D. L. Gal, E. Girard, T. Popova, P. L. Rosa, J. Beauvallet, S. Eon-Marchais, M.-G. Dondon, C. D. d’Enghien, A. Laugé, W. Chemlali, V. Raynal, M. Labbé, I. Bièche, S. Baulande, J.-O. Bay, P. Berthet, O. Caron, B. Buecher, L. Faivre, M. Fresnay, M. Gauthier-Villars, P. Gesta, N. Janin, S. Lejeune, C. Maugard, S. Moutton, L. Venat-Bouvet, H. Zattara, J.-P. Fricker, L. Gladieff, I. Coupier; CoF-AT; GENESIS, kConFab; G. Chenevix-Trench, J.

Hall, A. Vincent-Salomon, D. Stoppa-Lyonnet, N. Andrieu, F. Lesueur, Morphology and genomic hallmarks of breast tumours developed by ATM deleterious variant carriers. *Breast Cancer Res.* **20**, 28 (2018).

85. W. R. Bodily, B. H. Shirts, T. Walsh, S. Gulsuner, M.C. King, A. Parker, M. Roosan, S. R. Piccolo, Effects of germline and somatic events in candidate BRCA-like genes on breast-tumor signatures. *PLOS ONE* **15**, e0239197 (2020).

86. T. C. G. A. Network, Comprehensive molecular portraits of human breast tumours. *Nature* **490**, 61–70 (2012).

87. E. Cerami, J. Gao, U. Dogrusoz, B. E. Gross, S. O. Sumer, B. A. Aksoy, A. Jacobsen, C. J. Byrne, M. L. Heuer, E. Larsson, Y. Antipin, B. Reva, A. P. Goldberg, C. Sander, N. Schultz, The cBio Cancer Genomics Portal: An open platform for exploring multidimensional cancer genomics data. *Cancer Discov.* **2**, 401–404 (2012).

88. C. A. Miller, Y. Gindin, C. Lu, O. L. Griffith, M. Griffith, D. Shen, J. Hoog, T. Li, D. E. Larson, M. Watson, S. R. Davies, K. Hunt, V. J. Suman, J. Snider, T. Walsh, G. A. Colditz, K. DeSchryver, R. K. Wilson, E. R. Mardis, M. J. Ellis, Aromatase inhibition remodels the clonal architecture of estrogen-receptor-positive breast cancers. *Nat. Commun.* **7**, 12498 (2016).

89. The Metastatic Breast Cancer Project. https://mbcproject.org/data-release.

90. S. P. Shah, A. Roth, R. Goya, A. Oloumi, G. Ha, Y. Zhao, G. Turashvili, J. Ding, K. Tse, G. Haffari, A. Bashashati, L. M. Prentice, J. Khattra, A. Burleigh, D. Yap, V. Bernard, A. McPherson, K. Shumansky, A. Crisan, R. Giuliany, A. Heravi-Moussavi, J. Rosner, D. Lai, I. Birol, R. Varhol, A. Tam, N. Dhalla, T. Zeng, K. Ma, S. K. Chan, M. Griffith, A. Moradian, S. W. G. Cheng, G. B. Morin, P. Watson, K. Gelmon, S. Chia, S.F. Chin, C. Curtis, O. M. Rueda, P. D. Pharoah, S. Damaraju, J. Mackey, K. Hoon, T. Harkins, V. Tadigotla, M. Sigaroudinia, P. Gascard, T. Tlsty, J. F. Costello, I. M. Meyer, C. J. Eaves, W. W. Wasserman, S. Jones, D. Huntsman, M. Hirst, C. Caldas, M. A. Marra, S. Aparicio, The clonal and mutational evolution spectrum of primary triple-negative breast cancers. *Nature* **486**, 395–399 (2012).
